# A Multi-level ensemble approach for skin lesion classification using Customized Transfer Learning with Triple Attention

**DOI:** 10.1371/journal.pone.0309430

**Published:** 2024-10-24

**Authors:** Anwar Hossain Efat, S. M. Mahedy Hasan, Md. Palash Uddin, Md. Al Mamun

**Affiliations:** 1 Department of Computer Science and Engineering, Rajshahi University of Engineering & Technology, Rajshahi, Bangladesh; 2 Department of Computer Science and Engineering, Hajee Mohammad Danesh Science and Technology University, Dinajpur, Bangladesh; Manipal Institute of Technology, Manipal Academy of Higher Education, INDIA

## Abstract

Skin lesions encompass a variety of skin abnormalities, including skin diseases that affect structure and function, and skin cancer, which can be fatal and arise from abnormal cell growth. Early detection of lesions and automated prediction is crucial, yet accurately identifying responsible regions post-dominance dispersion remains a challenge in current studies. Thus, we propose a Convolutional Neural Network (CNN)-based approach employing a Customized Transfer Learning (CTL) model and Triple Attention (TA) modules in conjunction with Ensemble Learning (EL). While Ensemble Learning has become an integral component of both Machine Learning (ML) and Deep Learning (DL) methodologies, a specific technique ensuring optimal allocation of weights for each model’s prediction is currently lacking. Consequently, the primary objective of this study is to introduce a novel method for determining optimal weights to aggregate the contributions of models for achieving desired outcomes. We term this approach “Information Gain Proportioned Averaging (IGPA),” further refining it to “Multi-Level Information Gain Proportioned Averaging (ML-IGPA),” which specifically involves the utilization of IGPA at multiple levels. Empirical evaluation of the HAM1000 dataset demonstrates that our approach achieves 94.93% accuracy with ML-IGPA, surpassing state-of-the-art methods. Given previous studies’ failure to elucidate the exact focus of black-box models on specific regions, we utilize the Gradient Class Activation Map (GradCAM) to identify responsible regions and enhance explainability. Our study enhances both accuracy and interpretability, facilitating early diagnosis and preventing the consequences of neglecting skin lesion detection, thereby addressing issues related to time, accessibility, and costs.

## 1 Introduction

Skin lesions encompass atypical changes in the skin’s appearance, while skin diseases encompass a spectrum of conditions affecting the skin’s health, structure, and functionality [1]. These conditions range from common problems like acne to more critical issues such as skin cancer. Skin diseases may manifest with a variety of symptoms and are not exclusively defined by the presence of lesions. Skin lesions can occur due to infections, inflammatory conditions, allergic reactions, skin cancer, insect bites, trauma, autoimmune diseases, genetic factors, environmental factors, vascular abnormalities, warts, and cysts, with each category having various specific causes and characteristics [2]. Skin lesions can be categorized into two broad types based on their potential for harm: ‘Benign skin lesions’ are non-cancerous and generally harmless, including moles, skin tags, warts, seborrheic keratoses, and hemangiomas, while ‘Malignant skin lesions’ are cancerous lesions with the potential to spread to other parts of the body, such as basal cell carcinoma, squamous cell carcinoma, and melanoma.

Effective diagnosis and treatment typically necessitate a combination of clinical assessments and diagnostic tests. Neglecting symptoms can result in grave repercussions, including the development of skin cancer, which is the most prevalent form of cancer worldwide [3]. Among skin cancers, melanoma, albeit relatively infrequent, accounts for the majority of skin cancer-related fatalities. According to the National Cancer Society, in 2023, there were approximately 97,610 new cases of melanoma of the skin in the United States, with a death toll of 7,990 individuals. Additionally, it is estimated that approximately 2.2 percent of both men and women will be diagnosed with melanoma of the skin at some point during their lifetime. In 2020, there were an estimated 1,413,976 individuals living with melanoma of the skin in the United States, highlighting the significant impact of this disease on the population.

Early identification of skin lesions is of paramount significance. However, many individuals may lack awareness due to the extensive array of medical evaluations required, coupled with the associated financial burdens [4]. Dermatoscopy, also referred to as dermoscopy or epiluminescence microscopy, is a non-invasive diagnostic technique in dermatology that employs a specialized handheld device with magnification and lighting to assess skin lesions, facilitating early detection of skin cancer and other dermatological conditions in traditional detection systems. However, complete dependency on expert doctors may lead to human errors. Early identification of skin lesions is of paramount significance. However, many individuals may lack awareness due to the extensive array of medical evaluations required, coupled with the associated financial burdens. Dermatoscopy, also referred to as dermoscopy or epiluminescence microscopy, is a non-invasive diagnostic technique in dermatology that employs a specialized handheld device with magnification and lighting to assess skin lesions, facilitating early detection of skin cancer and other dermatological conditions in traditional detection systems. However, complete dependency on expert doctors may lead to human errors.

Conversely, an automated system empowered by AI, particularly leveraging ML and DL techniques, has the potential to detect skin lesions by analyzing a constrained dataset of images. Such a system can considerably expedite early diagnosis, thereby augmenting awareness about the condition and potentially yielding more efficacious medical interventions [5]. Numerous researchers have delved into the utilization of ML and DL methodologies. Nonetheless, there remains substantial room for enhancement in this domain. One pivotal consideration is the adept training of models to mitigate undue dependence on classes abundant in data. The direct application of models rooted in TL, pre-trained on the ImageNet dataset, can falter in extracting shallow features, rendering them ill-suited for specific datasets unless meticulously fine-tuned. Some methods rely on combining different models, but deciding how much each model should contribute can be challenging and affect the overall performance. The current state of models also does not prioritize making the results easy to understand. Additionally, using the same data for both validation and testing can introduce bias and impact the accuracy of model evaluation.

Within our exploration, our methodology is strategically designed to directly confront these limitations. Besides this, the paramount objective is centered around addressing the fundamental research questions enumerated below, while the formulation of a robust architectural framework is underpinned by providing suitable responses based on them. These inquiries are prerequisites, demanding comprehensive answers.

RQ1: *What actions can be implemented to achieve a balanced distribution of classes and thus generate an optimal dataset for training?* As the number of samples in each class varies, it is possible for the majority classes to dominate, thereby hindering the accurate prediction of the minority. Hence the distribution of the classes should be well-balanced.

RQ2: *What approach can be utilized to highlight the most critical features, more specifically, significant areas or regions?* Some regions of an image may not be important for feature extraction in a classification problem due to redundant or irrelevant information with a negative impact, while others may be more significant in indicating the target class.

RQ3: *Is the use of a single algorithm adequate or are additional EL techniques required, and if so, which one should be employed?* Since not all algorithms possess the capability to accurately classify all data, it is imperative to diminish the reliance on a single algorithm. So, An EL technique can be the most suitable solution.

RQ4: *What are the limitations of traditional EL methods that necessitate the introduction of a new EL approach?* Since no technique can ensure the definitive division of the optimal ratio for each model’s prediction, introducing a new approach that can calculate the optimal ratio of the predictions and then ensemble the models is necessary. The aforementioned inquiries are meticulously addressed, and our study has culminated with the subsequent contributions.

- The challenge of class imbalance is effectively addressed by rigorously augmenting the training dataset. This strategic augmentation is executed while maintaining a balanced distribution across classes, ensuring that favoritism towards dominant classes is not exhibited by the model. Consequently, reliability is instilled, and impartiality of test and validation data is demonstrated by our architecture.
- To ensure that adequate attention is given to crucial features, a remarkable methodology is ingeniously incorporated: the integration of TA mechanisms within individually customized architectures. This innovative approach empowers models to be focused on the most pivotal aspects of the input data.
- To extract both shallow and deep features, which are more complex to extract, Customized Transfer Learning (CTL) models, are meticulously originated by seamlessly integrating them with customized CNN-based approaches. This fusion allows the harnessing of the strengths of both paradigms, resulting in a comprehensive feature extraction process.
- A pioneering technique, IGPA, is introduced to elevate the robustness, accuracy, and generalization capabilities of the model. This novel Ensemble technique operates across multiple levels, strategically enhancing the performance of the architecture.
- The interpretability of the architecture is prioritized by incorporating GradCAM visualization. This advanced visualization method empowers the model to have specific regions highlighted for the diagnosed skin conditions, enhancing the architecture’s transparency and insightfulness.

The structure of the paper is meticulously organized to ensure clarity and coherence. It commences with an in-depth exploration of the existing literature in Section 2, followed by a comprehensive presentation of the materials and methods in Sections 3 and 4. The subsequent section, Section 5, provides a concise yet thorough analysis of the achieved performances. Building on these findings, Section 6 engages in a comprehensive discourse, assessing the model’s pragmatic implications. The study’s limitations are thoughtfully outlined in Section 7, offering a holistic view. Ultimately, Section 8 concludes the paper, encapsulating the essential takeaways and contributions of the study.

## 2 Literature review

The field of skin lesion classification has been extensively investigated by numerous researchers, who dedicated their efforts to uncover the intricate complexities within this domain. In this section, we embark on a journey to shed light on the diverse contributions made by these scholarly endeavors. Studies [6] through [10] introduced various classification and segmentation methods, each offering unique insights that inspire our current research endeavor. From the work of study [11] to study [16], a Custom CNN architecture was employed, among them, study [14] to study [16] integrated various types of transformation processes. Thinking creatively, studies [17, 18] employ some unique and innovative methods on skin lesion datasets. In contrast, studies [19] to [26] focused on feature extraction using Transfer Learning (TL), while studies [27, 28] utilized soft attention in conjunction with TL.

The study by Tajerian et al. [6] introduced a methodological approach utilizing transfer learning with EfficientNET-B1, achieving an accuracy of 84.30% in diagnosing pigmented skin lesions. However, its limitation lies in the inability of the EfficientNET architecture to emphasize specific features unique to the skin dataset, potentially impacting diagnostic precision in certain cases. The SkiNet framework proposed by [7] utilized Bayesian MultiResUNet for segmentation and DenseNet-169 for classification, significantly enhancing skin lesion classification accuracy of 86.67% which can’t be considered as a satisfactory performance. The introduction of the SkinViT architecture by [8], incorporating an outlook attention mechanism, transformer block, and MLP head block, achieving a maximum accuracy of 91.09% on a different dataset, thus significantly improving the classification of Melanoma and Nonmelanoma skin cancers. Hosny et al. [9] presented an automatic skin lesions classification system with higher accuracy rates achieved through transfer learning with AlexNet, offering improved efficiency in diagnosing melanoma and nevus lesions. Dong et al. [10] introduced TC-Net, a dual coding fusion network combining Transformer and CNN architectures, significantly improving skin lesion segmentation performance by effectively integrating local and global feature information, outperforming single-network models such as Swin UNet by notable margins across multiple datasets.

In a study by Shetty et al. [11], a CNN was employed to detect skin cancer, yielding an accuracy of 94%. However, their approach had a limitation in that it utilized only a subset of the dataset (200 images per class), augmenting it, which raises concerns about the validity of applying the results to the entire dataset. Sevli [12] developed a CNN model for classifying skin lesions, integrating it with a web application via a REST API. The model underwent evaluation by dermatologists in two phases, achieving an accuracy of 91.51%. Notably, their custom CNN design could not focus on crucial features. In an alternate strategy, Saarela and Geogieva [13] introduced a novel approach based on Bayesian inference to enhance model interpretability, demonstrating its effectiveness. However, their achieved accuracy of 80% on their test data falls short of being particularly promising.

Nie et al. [14] put forth a hybrid CNN transformer model enhanced with focal loss for skin lesion classification, attaining an accuracy of 89.48%. Their approach combined a CNN for extracting low-level features and a vision transformer though there exists a limitation of extracting deep features. Hoang et al. [15] introduced an innovative segmentation technique and utilized the lightweight neural network architecture, wide-ShuffleNet, for skin lesion classification which results in comparatively lower accuracy. Their achieved accuracies were 84.80% and 86.33% on different sizes of test data. In another study by Sun et al. [16], a model was proposed that incorporated additional metadata and integrated supplementary information during the data augmentation process. The approach yielded an accuracy of 88.7% with a single model and 89.5% for the embedding solution. The augmentation process was not described in a well-interpretable way.

The study by Ajmal et al. [17] proposes a novel architecture for multiclass skin lesion classification that combines deep learning models (Inception-ResNetV2 and NasNet Mobile), fuzzy entropy slime mould algorithm for feature optimization, and Serial-Threshold fusion for feature integration, achieving superior accuracy on HAM10000 and ISIC 2018 datasets employing Grad-CAM for explainability, resulting in satisfactory classification accuracies. Another work by Khan et al. [18] introduces a novel deep learning and Entropy-NDOELM-based architecture for multiclass skin lesion classification, addressing limitations in accuracy and computational efficiency. The method involves contrast enhancement, fine-tuning EfficientNetB0 and DarkNet19 models, feature extraction and selection via Entropy-NDOELM, feature fusion, and final classification using an extreme learning machine, achieving good performances of more than 90% in all datasets.

Mahbod et al. [19] investigated the impact of image size on skin lesion classification. Their study utilized TL techniques and highlighted the success of a multi-CNN fusion approach, achieving a balanced multi-class accuracy of 86.2% which is not so promising though the model was comparatively heavy. Rahman et al. [20] devised a weighted average ensemble learning model that harnessed five deep neural network models via TL. This ensemble approach notably enhanced the results, leading to an impressive 88% accuracy. Due to the direct use of the pre-trained model, they could not cope with the model with the specific dataset. Wang et al. [21] introduced a unique two-stream network named the feature fusion module, which cleverly combined DenseNet-121 and VGG-16. This fusion aimed to extract multiscale pathological information using multi-receptive fields and GeM pooling to curtail the spatial dimensionality of lesion features. This innovative approach yielded an elevated test accuracy of 91.24% though there was a lack of fine tuning the pre-trained model. Harangi et al. [22] proposed a Transfer Learning-based CNN framework for multiclass classification using binary classification outcomes. Their study revealed that incorporating binary classification results led to a substantial improvement in accuracy of an average of 93.46% for the multi-class problem, a notable increase of 7%. Their approach of combining the binary classification with multi-class did not contain the justification.

Khan et al. [23] employed Resnet50 and a feature pyramid network for skin lesion segmentation, followed by a 24-layered CNN for classification, resulting in an accuracy of 86.5%. However, their approach omitted the utilization of mask information from the classification dataset (HAM10000) during the segmentation phase. Popescu et al. [24] devised a skin lesion classification system that harnessed various Transfer Learning (TL) techniques, coupled with collective intelligence. Their methodology achieved a validation accuracy of 86.71% through a decision fusion module. Notably, no results were provided for an independent test dataset. Gouda et al. [25] enhanced the quality of skin lesion images using ESRGAN before applying a CNN, leading to an accuracy of 83.2%. Despite experimenting with several transfer learning models, their study did not address the challenge of imbalanced data. Nigar et al. [26] introduced an explainable AI-based skin lesion classification system, leveraging the LIME framework and ResNet-18. This approach achieved notable accuracy (94.47%) and interpretability, aiding early-stage skin cancer diagnosis. Limitations include reliance on a single pre-trained model, a small dataset, and potential downsizing effects on image pre-processing.

Nguyen et al. [27] introduced an innovative method that combined deep learning with Soft-Attention. They obtained a 90% accuracy using InceptionResNetV2 and an 86% accuracy using MobileNetV3Large. They did not clarify the reason for using soft attention instead of other modules. Datta et al. [28] investigated the impact of the Soft-Attention mechanism in skin cancer classification, aiming to boost model performance. Their work surpassed state-of-the-art precision and AUC scores on two datasets, reaching an impressive accuracy of 93.4%. This model holds potential for aiding dermatologists in dermoscopy systems but could not be able to find proper color channel weights of attention.

Drawing upon the insights from the aforementioned literature, certain limitations are identified and addressed in our research. Specifically, the entire dataset is utilized for the investigation, with a focus on augmenting the training set to rectify the issue of data imbalance. This ensures the independence of the test set for a more accurate model evaluation of unseen data. The crucial regions of interest are pinpointed by harnessing the TA method and seamlessly integrating it with TL models. Furthermore, following the extraction of intricate features, the TL models and CTL architecture are fine-tuned, mitigating the overreliance on the ImageNet dataset.

## 3 Dataset

Our study utilizes the publicly available Human Against Machine (HAM10000) dataset from the Harvard Dataverse repository, meticulously curated to encompass a diverse collection of skin lesion samples [29]. It includes 10,015 dermatoscopic images, all in jpg format, distributed into 7 classes: Melanoma (MEL), Nevus (NV), Vascular lesions (VASC), Actinic keratosis (AK), Basal Cell Carcinoma (BCC), Benign keratosis (BKL), and Dermatofibroma (DF), where MEL, AK, and BCC are types of cancer [30]. NV, BKL, and DF are non-cancerous, whereas some types of VASC can be cancerous. The overview of the dataset is presented in Table 1.

**Table 1:** Portrayal of the dataset.

No of images	Format	No of classes	Source
10015	JPG	7	Harvard Dataverse

**Fig 1:**
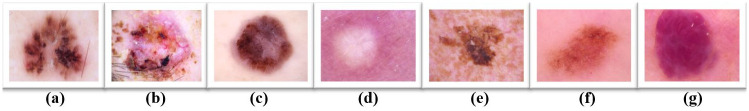
Sample Images of Different Classes (a) Actinic Keratosis (b) Basal Cell Carcinoma (c) Benign Keratosis (d) Dermatofibroma (e) Melanoma (f) Nevus (g) Vascular Lesions.

**Fig 2:**
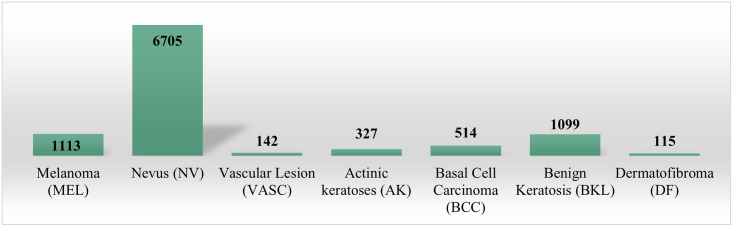
Instance distribution for each class.

In Fig 1, examples of images are displayed, with one sample provided per class in the dataset, while the high degree of class representation imbalance is corroborated by the class distribution depicted in Fig 2.

## 4 Methods and materials

**Fig 3:**
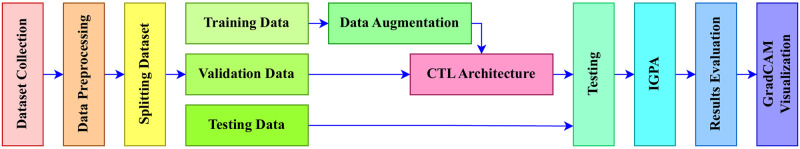
Schematic representation of methodology.

Our methodology commences with the collection of a dataset, a step that is followed by a crucial process known as data preprocessing. Subsequently, the dataset is divided into training, testing, and validation subsets. To address class imbalances, the augmentation process takes place exclusively on the training data. This ensures that our study’s validation is conducted independently on unseen testing and validation data. CTL architectures are then employed, which have been fitted using the training data and validated using the validation data. The performance of these fitted models has been assessed using the testing data. Predictions generated by each architecture are combined through IGPA to enhance performance. The evaluation of IGPA is carried out on multiple levels to substantiate our claims. Finally, the GradCAM visualization technique is employed to elucidate the internal capabilities of the models. The sequential workflow of this exploration is depicted in Fig 3.

### 4.1 Preprocessing and data augmentation

In this phase, we begin by organizing the images according to their lesion IDs, then selectively sample distinct images for the training, testing, and validation sets. Specifically, we allocate 15% of the images to both the testing and validation datasets, leaving 70% for training purposes. We also introduce additional redundant images into the training set to ensure that the test set consists of entirely unseen images, thereby enhancing the robustness and credibility of our model. This separation ensures that the testing data remains completely unseen during training. Following this, we apply augmentation techniques exclusively to the training data to maintain the independence of the test and validation sets. This strategy generates around 8000 images per class, effectively addressing potential data imbalance issues.

**Fig 4:**
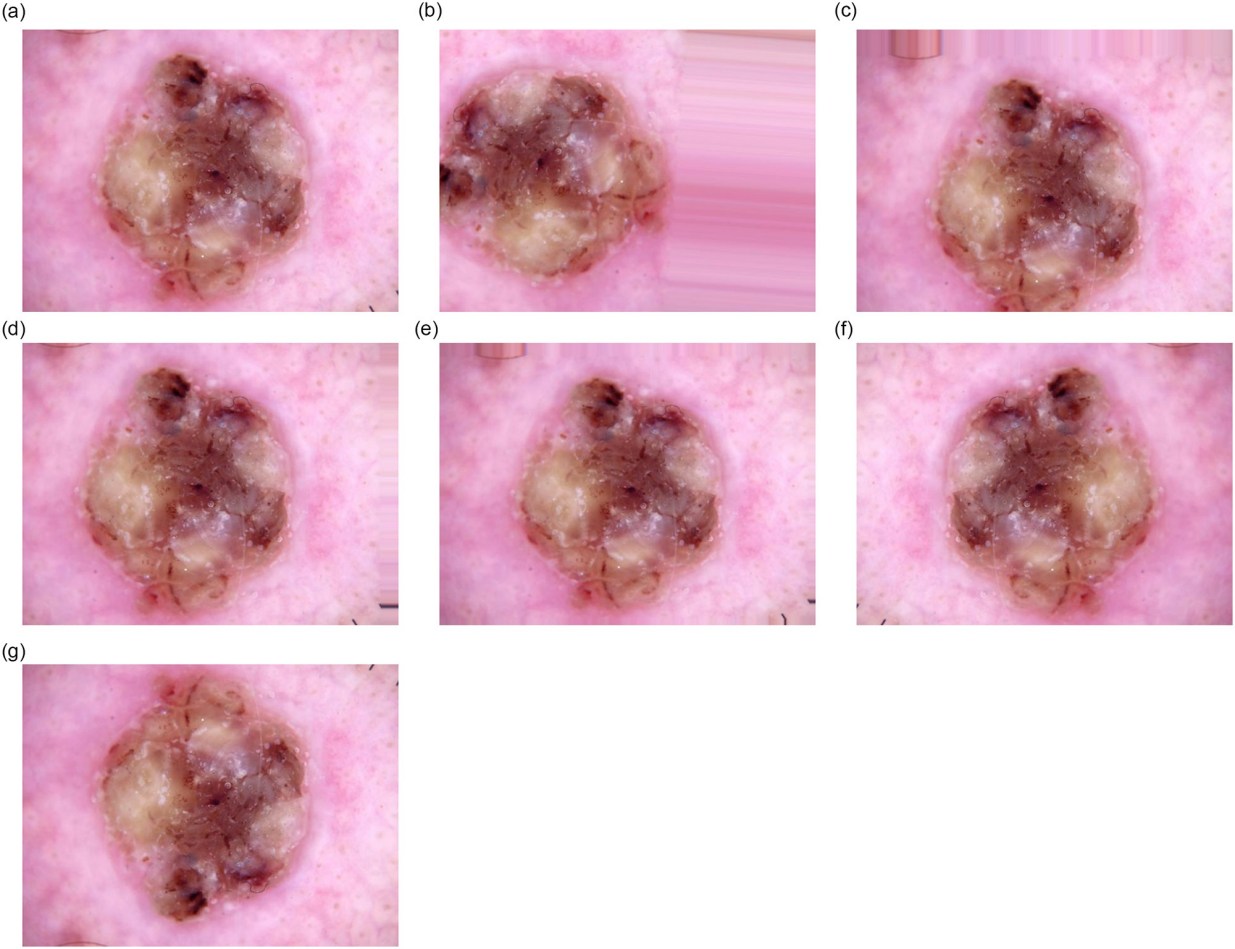
Sample images of the images after augmentation. (a) Original Sample, (b) Rotated Sample, (c) Width_shifted, (d) Height_shifted, (e) Zoomed, (f), Horizontal_flipped, (g) Vertical_flipped.

In our research, we employed an advanced image augmentation strategy using TensorFlow’s “ImageDataGenerator”. We started by enhancing the contrast of the original images to ensure optimal quality before augmentation. The augmentation process involved a variety of transformations to significantly diversify the training data and enhance the model’s robustness. We applied random rotations up to 180 degrees, width and height shifts of 10%, and zoom variations within a 10% range. Additionally, horizontal and vertical flips were used to increase variability. To handle gaps introduced by these transformations, we used the nearest neighbor fill mode, ensuring coherence in the augmented images. This comprehensive approach simulates a wide range of possible image variations, thereby improving the generalization capability of our deep learning model. Fig 4 illustrates the original, contrast-enhanced, and augmented images, using a sample from Actinic keratosis (AK) and its augmented versions. However, to address the issue of an imbalanced dataset, our goal was to generate approximately 8000 images in the training set for each class. Consequently, we achieved the following distribution of images: AK (7854), BCC (7965), BKL (7944), DF (7377), MEL (7932), NV (8004), and VASC (7706).

### 4.2 Creation of CTL architectures

**Fig 5:**
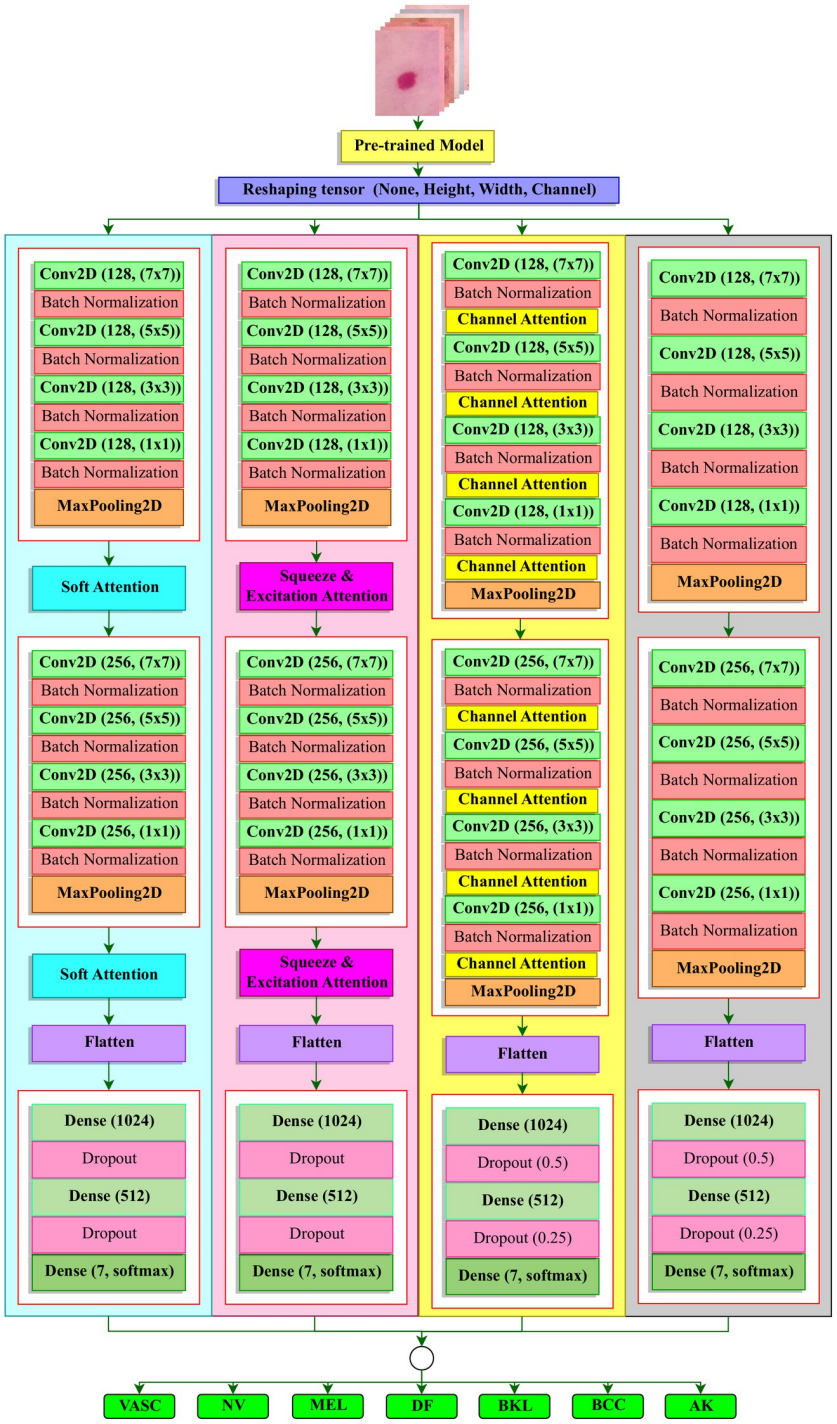
CTL architecture.

Our primary focus is centered around the utilization of customized pre-trained models to effectively leverage the principles of TL. This is initiated by leveraging the saved weights of pre-trained models. For precision, our study utilizes a set of 9 pre-trained models to create CTL architectures. These include variants of DenseNet (DenseNet121, DenseNet169, DenseNet201) and MobileNet (MobileNet, MobileNetV2, MobileNetV3Large), all of which accept input images of size (224x224x3). Additionally, InceptionV3, InceptionResnetV2, and Xception models are employed, which require input images of size (299x299x3). Since these weights were not originally trained for our dataset, we engage in the process of fine-tuning them so that they can extract both shallow and deep features for our dataset. This fine-tuning is carried out using four fundamental CNN structures: Customized Convolutional Neural Network (CCNN), Channel Attention-based Convolutional Neural Network (CACNN), Squeeze and Excitation Attention-based Convolutional Neural Network (SEACNN), and Soft Attention-based Convolutional Neural Network (SACNN), all of which have been uniquely created by us with various combinations of Triple Attention. The graphical insight of the complete architecture is depicted in Fig 5. Detailed explanations of the models inside the architecture are meticulously provided in the upcoming paragraphs.

#### 4.2.1 Customized Transfer Learning (CTL) models with basic fine tuning blocks

In the integration of the pre-trained models with our CCNN, CACNN, SEACNN, or SACNN, the procedure is initiated by importing the pre-trained model from the ‘keras’ library. Subsequently, the model is instantiated with our unique input shape and transformed its output into a four-dimensional structure: None, height, width, and the number of channels. This adaptation is necessary to align our model with the pre-trained one, as our model requires a four-dimensional input while the pre-trained model’s output tensor contains only two dimensions.

Following this, the process of fine-tuning is initiated and executed in a step-by-step manner. The culmination of this process involves recording predictions from each individualized model for subsequent analysis.

#### 4.2.2 Organization of basic fine tuning blocks by customized CNN with Triple Attention

The structure of our CCNN consists of two sets of Convolution Blocks, each featuring different quantities of filters. Each Block integrates four ‘Conv2D’ layers with various kernel sizes: (7x7), (5x5), (3x3), and (1x1), accompanied by corresponding ‘BatchNormalization’ layers. This is followed by a ‘MaxPooling2D’ layer that condenses the output. The initial block comprises 128 filters, while the subsequent one embraces 256 filters. In all convolutional layers, the ‘ReLU’ activation function is utilized due to its effectiveness in managing the challenge of vanishing gradients.

To utilize the Channel Attention (CA) module in CACNN, the integration of this CA Layer within each convolution block as detailed in the earlier paragraph is executed which illustrates the inclusion of the CA layer after each ‘Conv2D’ layer along with its corresponding ‘Batch Normalization’ layer. The positioning of the CA Layer between consecutive ‘Conv2D’ layers aims at feature refinement at an intermediate stage of convolutional processing. This arrangement allows selective emphasis on significant channel-wise information and the suppression of less relevant details before further processing. In implementing the Squeeze and Excitation Attention (SEA) module to create SEACNN, the embedding of the SEA Layer after each convolution block follows the same approach outlined in the CCNN organization. The SEA Layer, integrated after each convolution block, focuses on recalibrating the feature responses across all channels. Its placement here allows for high-level adjustment of channel-wise importance after multiple convolutional operations, enhancing the model’s capability to capture complex and hierarchical features.

To incorporate the Soft Attention (SA) module to organize SACNN, the SA Layer is included similarly to the SEACNN approach, positioned after each convolution block. The placement of SA layers after each block allows the model to capture fine-grained patterns within the feature maps. However, due to the increased number of parameters within the internal organization when SA is added, this layer is not utilized after each ‘Conv2D’ layer. The output derived from the final max-pooling layer from every architecture is flattened and directed into a sequence of three fully connected layers. Within this configuration, a singular fully connected block is introduced, utilizing three ‘Dense’ layers with tensor dimensions of 1024, 512, and 7 (corresponding to the number of classes). The initial two fully connected layers also employ the ‘ReLU’ activation function, while the concluding layer incorporates the ‘softmax’ activation function to anticipate class probabilities.

Additionally, an extra layer of sophistication is introduced into the initial two dense layers through the inclusion of ‘Dropout’ mechanisms, which serve to deter overfitting and regularization. This process begins with a dropout rate of 50% in the first layer, followed by a 25% rate in the subsequent one.

#### 4.2.3 Feature extraction process

We utilized a Transfer Learning model pre-trained on ImageNet for feature extraction, excluding its top fully connected layers (include_top = False) and applying global average pooling (pooling=‘avg’). The model’s output was reshaped to dimensions (8, 8, 26) before passing through multiple convolutional layers with filter sizes of 7x7, 5x5, 3x3, and 1x1, each followed by ReLU activation and batch normalization for stabilization. Max pooling layers were employed to reduce spatial dimensions and enhance feature focus. The flattened feature maps were processed through fully connected layers with ReLU activation, culminating in a dense layer with softmax activation for class probability distribution.

**Fig 6:**
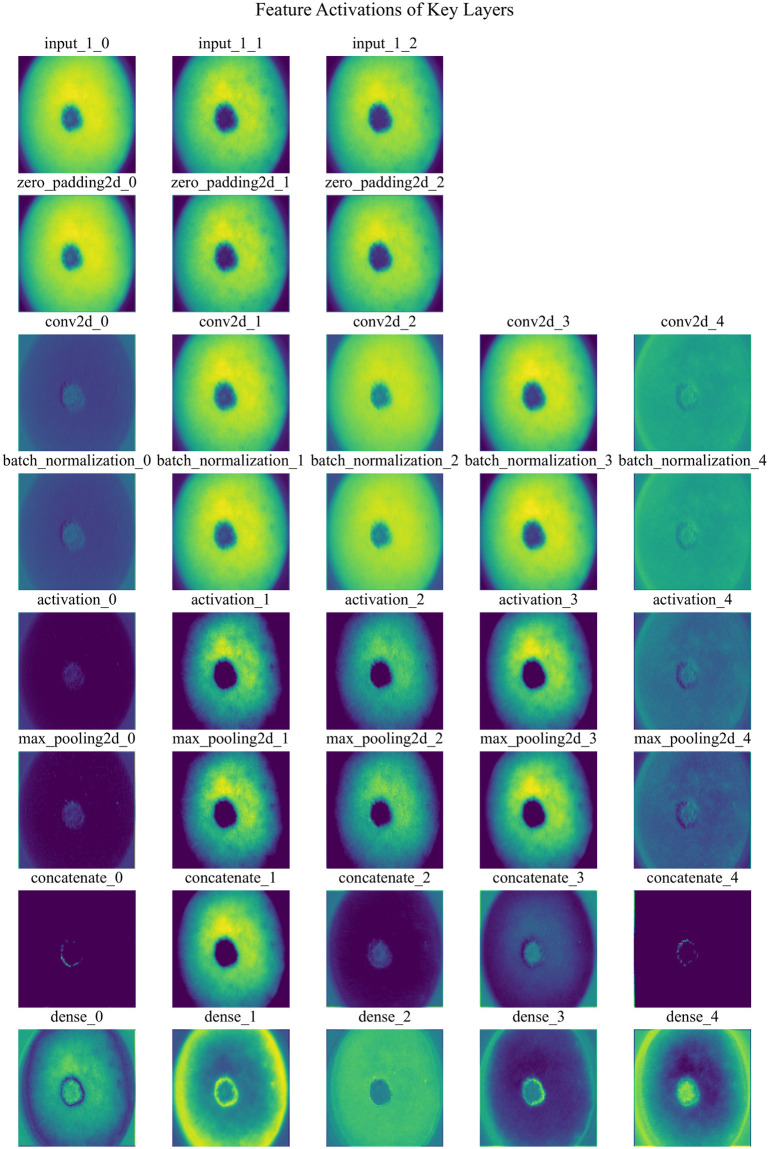
Feature extraction after activation of each layer (One image as example).

Fig 6 illustrates the feature map activations at different layers of a Transfer Learning model, specifically an example of a modified DenseNet169 architecture. Each row corresponds to activations from a distinct layer in the model.

Input Layer (input_1): The initial input image after preprocessing, showing the raw pixel data.

Zero Padding (zero_padding2d): Feature maps after applying zero padding to the input tensor, preparing it for convolution operations.

Convolution (conv2d): Activation maps after passing through a convolutional layer with 64 filters, highlighting learned patterns and edges.

Batch Normalization (batch_normalization): Normalized feature maps following batch normalization, enhancing training stability and convergence.

ReLU Activation (activation): Output after applying rectified linear unit (ReLU) activation function, introducing non-linearity to the network.

Max Pooling (max_pooling2d): Downsampled feature maps post max pooling, reducing spatial dimensions while retaining important features.

Concatenation (concatenate): Activation maps after concatenating feature maps from previous layers, integrating information from multiple paths.

Dense Layer (dense): Feature maps are transformed into a vector representation before entering the fully connected dense layer.

Output Layer (dense_1): Final layer activations depicting class probabilities through a softmax activation function.

Each subplot displays up to 7 filters per layer, visualized using the ‘viridis’ colormap for clarity. The figure provides insights into how the model processes and transforms input images through successive layers, capturing hierarchical features crucial for classification tasks.

This exemplifies a single sample and a subset of layers. Through this approach, we have extracted thousands of feature images that significantly enhance algorithm performance.

### 4.3 Triple Attention (TA)

Our study employs three attention modules to highlight crucial input features and disregard irrelevant ones.

#### 4.3.1 Channel Attention (CA)

CA improves feature maps by calculating channel-wise attention weights using mean and standard deviation. These weights are applied to input feature maps to emphasize essential features [31].

$$\begin{array}{c} {\text{w}}_{\text{c}}=\sigma ({\text{W}}_{2}\delta ({\text{W}}_{1}\text{x})) \end{array}$$ (1)

$$\begin{array}{c} {\text{y}}_{\text{c}}={\text{w}}_{\text{c}}⊙\text{x}, \end{array}$$ (2)

where, **x** = input feature maps *C* × *H* × *W*, **W**_**1**_, **W**_**2**_ = weight matrices; *δ* = ReLU activation; *σ* = sigmoid activation; **w**_**c**_ = calculated attention weights; and ⊙ represents element-wise multiplication [31].

#### 4.3.2 Squeeze and Excitation Attention (SEA)

The SEA module combines a spatial dimension reduction operation and channel-wise attention learning [32]. Let **x** be input feature maps of size *C* × *H* × *W*. Then,

$$\begin{array}{c} \text{z}=\text{GlobalAvgPooling}(\text{x}) \end{array}$$ (3)

$$\begin{array}{c} \text{s}=\text{ReLU}({\text{W}}_{2}\text{sigmoid}\left({\text{W}}_{1}\text{z}\right)) \end{array}$$ (4)

$$\begin{array}{c} \text{y}=\text{s}⊙\text{x}. \end{array}$$ (5)

#### 4.3.3 Soft Attention (SA)

SA assigns weights to input elements, focusing on particular portions based on their relative importance [28] as follows.

$$\begin{array}{c} a_{i}=\frac{\text{exp}(e_{i})}{\sum_{j=1}^{T}\text{exp}\left(e_{j}\right)}, \end{array}$$ (6)

where *a*_*i*_ = attention weight for the *i*-th input element; *T* = input length, and *e*_*i*_ = scalar for the *i*-th element [28].

### 4.4 Information Gain Proportioned Averaging (IGPA)

The novel approach of ensemble learning, Information Gain Proportioned Averaging (IGPA), is introduced by us. It calculates the most suitable weights for predictions from each classifier and then combines them through averaging, considering these weights. To achieve this, the concept of information gain (IG) is employed. The sequential procedure for implementing IGPA is outlined as follows.

**Step—1** This method initiates by evaluating the information gained from predictions generated by individual classifiers. To achieve this, correctly classified samples are labeled as class ‘1’, while incorrectly classified ones are marked as ‘0’. Subsequently, IGPA computes the entropy of each prediction using the following process. Entropy is a measure of the uncertainty or randomness associated with a random variable *X*. It quantifies the amount of information needed to describe the outcomes of *X*. The entropy *H*(*X*) of a discrete random variable *X* with possible outcomes *x*_1_, *x*_2_, …, *x*_*n*_ and probabilities *p*(*x*_1_), *p*(*x*_2_), …, *p*(*x*_*n*_) is calculated using the following formula:

$$\begin{array}{c} H\left(X\right)=-\sum_{i=1}^{n=2}p\left(x_{i}\right){\text{log}}_{2}\left(p\left(x_{i}\right)\right), \end{array}$$ (7)

where *H*(*X*) represents the entropy of the random variable *X*; *p*(*x*_*i*_) represents the probability of event *x*_*i*_, the summation is taken over all possible outcomes *x*_*i*_; and the number of events n = 2. The entropy is maximal when all outcomes are equiprobable, and it decreases as the distribution becomes more concentrated on specific outcomes. Entropy is often used in information theory, machine learning, and decision-making to evaluate the unpredictability and information content of data.

**Step—2** The information gain (IG) is then calculated utilizing a distinct formula displayed below,

$$\begin{array}{c} IG=\alpha [Entropy\;before\;split-\sum_{i=1}^{n}\frac{|S_{i}|}{\left|S\right|}\cdot Entropy\left(S_{i}\right)], \end{array}$$ (8)

where Information Gain represents the measure of how much uncertainty or randomness is reduced after a split; Entropy before the split refers to the entropy of the target variable before deciding to split; *S*_*i*_ represents each subset; |*S*_*i*_| is the number of samples in subset *S*_*i*_; |*S*| is the total number of samples in the original set; *Entropy*(*S*_*i*_) is the entropy of subset *S*_*i*_; and *α* is the level of the ensemble.

#### 4.4.1 Significance of *α*

The parameter *α* introduced in our approach plays a significant role by determining how much emphasis is placed on each classifier during the ensemble process. This is done to ensure that the classifier with the highest accuracy is given more weight, making it the most influential component of the ensemble. The reason for this is to enhance the overall robustness and efficiency of the approach. To put it simply, think of it this way: imagine in the first level of our ensemble, one particular architecture performs significantly better than the others. We want to maintain this dominance in the subsequent levels because if it is not done, and all models have equal influence, the overall prediction quality may decrease. So, the use of the *α* parameter is necessary to adjust the influence of each model, effectively giving more importance to the models that provide higher Information Gain (IG). This ensures that models with more IG are more prominent as the ensemble levels increase, improving the overall performance of our approach. Since the value range of IG is from 0 to 1, this adjustment allows us to have more influence from models with higher IG based on the degree of their dominance.

**Step—3** After acquiring the information gain values for each classifier, the ensembling weights are calculated. These weights are determined based on the ratio of each classifier’s information gain to the total information gain. This weighting mechanism ensures that classifiers with higher information gains contribute more significantly to the ensemble.

$$\begin{array}{c} weight_{i}=\frac{IG_{i}}{\sum_{i=1}^{m}IG_{i}}, \end{array}$$ (9)

where *m* is the number of predictions.

**Fig 7:**
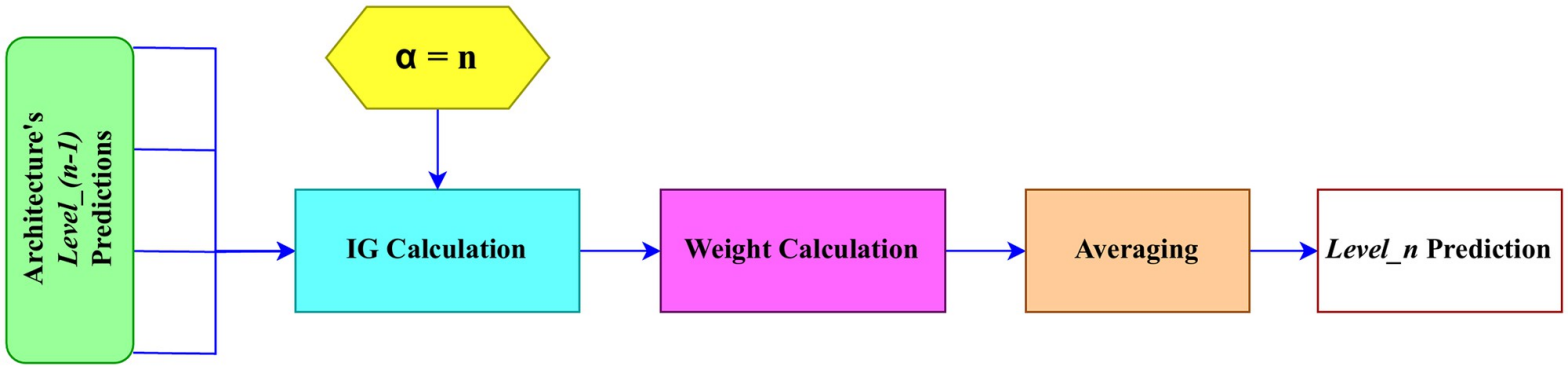
IGPA in Level—*n*.

**Step—4** Finally, the predictions are averaged according to the respective weights, yielding a blended result that capitalizes on the strengths of each classifier within the ensemble. This can be accomplished in the following way. Let *N* be the number of individual classifiers in the ensemble. Each classifier *i* produces predictions denoted as *P*_*i*_ = [*p*_*i*1_, *p*_*i*2_, …, *p*_*in*_], where *n* represents the number of instances in the dataset. The weights for each classifier, denoted as *w*_*i*_, are determined based on a specific criterion, such as accuracy or information gain. The ensemble prediction for each instance *j* is calculated as:

$$\begin{array}{c} E_{j}=\sum_{i=1}^{N}w_{i}\cdot p_{ij}, \end{array}$$ (10)

where *E*_*j*_ is the final prediction for instance *j*, *w*_*i*_ is the weight assigned to classifier *i*, and *p*_*ij*_ is the prediction of classifier *i* for instance *j*. The weights *w*_*i*_ are determined in a way that reflects the significance or performance of each classifier within the ensemble. This can be achieved through the previous step. Overall, the IGPA ensembling technique combines the predictions of individual classifiers using weighted averaging, where the weights are assigned based on their performance or relevance and determined by the concept of information gain. The overall approach of the IGPA method is illustrated in Fig 7.

### 4.5 Multi-Levelled IGPA

**Fig 8:**
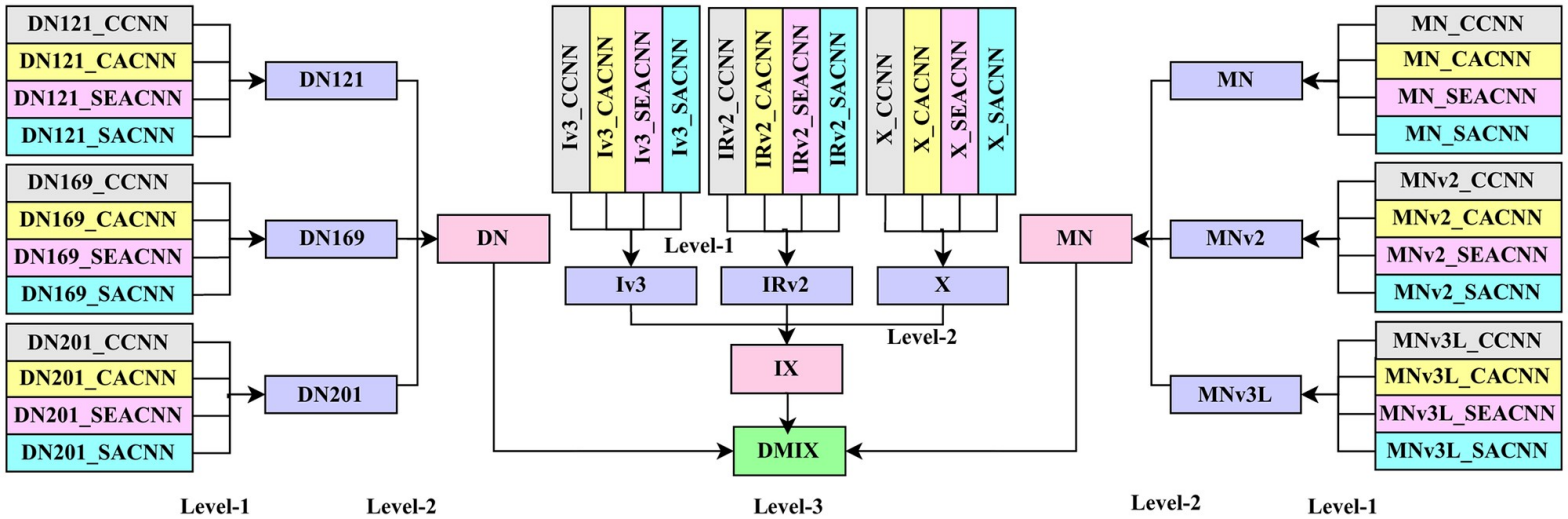
Organization of ML-IGPA.

Our IGPA technique is employed across multiple levels. This choice is made because, at a single level, it’s challenging to allocate sufficient emphasis to a specific superior model due to the low individual classifier weights. Consequently, a sequential “Level by Level” ensembling strategy is adopted. This approach allows accentuating different models that exhibit superior performance in comparison to others at each level. Subsequently, this emphasis is further compounded by subsequent ensembling of these optimized models in subsequent levels. The detailed explanation of our “Level by Level” approach is presented in the following sections, whereas the generic visual overview of ML-IGPA is demonstrated in Fig 8.

#### 4.5.1 IGPA in Level—1

At this stage, predictions obtained from the four fundamental models (CCNN, CACNN, SEACNN, and SACNN) for each classifier are combined through ensembling. Subsequently, a total of nine predictions are acquired from the first level through each of the CTL architectures. Importantly, for every individual prediction, both methodologies, including employing all predictions and selecting the best three predictions, are put into practice during this phase.

#### 4.5.2 IGPA in Level—2

The nine predictions derived from Level-1 are subsequently amalgamated through various combinations, resulting in three predictions at Level-2, i.e., DN, MN, and IX.

#### 4.5.3 IGPA in Level—3

The three predictions obtained from Level 2 are then combined to generate the ultimate level prediction, which serves as the outcome. Notably, both approaches involve the use of all previous-level predictions.

## 5 Experimental result analysis

### 5.1 Performance evaluation measures

In evaluating the performance and efficacy of our models, a variety of metrics including accuracy, precision, recall (sensitivity), f1-score, specificity, and the ROC-AUC (Receiver Operating Characteristic Area Under Curve) are employed, offering valuable insights into their predictive abilities. These metrics can all be derived from the confusion matrix, a summary table detailing the model’s predictions in terms of true positives, false positives, true negatives, and false negatives. The mathematical formulations of these measures are provided below.

$$\begin{array}{c} Accuracy=\frac{TP+TN}{TP+TN+FP+FN} \end{array}$$ (11)

$$\begin{array}{c} Precision=\frac{TP}{TP+FP} \end{array}$$ (12)

$$\begin{array}{c} Recall=\frac{TP}{TP+FN} \end{array}$$ (13)

$$\begin{array}{c} F1-Score=2\times \frac{Precision\times Recall}{Precision+Recall} \end{array}$$ (14)

$$\begin{array}{c} Specificity=\frac{TN}{TN+FP} \end{array}$$ (15)

$$\begin{array}{c} ROC\left(TPR\right)=\frac{TP}{TP+FN} \end{array}$$ (16)

$$\begin{array}{c} ROC\left(FPR\right)=\frac{FP}{FP+TN} \end{array}$$ (17)

By thoroughly assessing these metrics, we gain a comprehensive grasp of our models’ classification proficiency for skin lesions, enabling informed decisions regarding their real-world applicability.

### 5.2 Experimental setup

Our entire architecture is executed on a Kaggle notebook, leveraging the GPU P100 along with a 2-core Intel Xeon CPU, operating at a clock speed of 690 ms/step. Upon acquiring distinctive lesion images at a size of (224, 224, 3), the dataset is divided, reserving 15% for validation and an additional 15% for testing, while the remaining images are allocated for training purposes. The models undergo 50 epochs of training, utilizing a batch size of 16. The optimization process is driven by the Adam optimizer, initializing with a learning rate of 0.001. For loss computation, categorical cross-entropy is employed, and early stopping is implemented using Reduce on Plateau with a patience of 25. In this section, a comprehensive overview of both theoretical insights and graphical outcomes is offered to delve into the classification performances. Consequently, the primary aim of these results is to validate the effectiveness of employing IGPA as a means of enhancing performance. Through the presentation of experimental outcomes, which encompass an extensive quantity of evaluation metrics along with graphical representations of ROC-AUC curves and confusion matrices, a robust comparison of the various approaches delineated in preceding subsections is facilitated. The utilization of IGPA has been approached from three distinct perspectives: Multi-Levelled IGPA utilizing all classifiers at each level (*ML* − *IGPA*_*a*_), Multi-Levelled IGPA employing the top three classifiers at each level (*ML* − *IGPA*_*b*_), and Single-Level IGPA integrating all classifiers (*SL* − *IGPA*).

#### 5.2.1 Trainable parameters

Since we have ensemble the algorithms at the prediction level, the number of trainable parameters remains unchanged post-ensemble. Table 2 provides a summary of the trainable parameters.

**Table 2:** Trainable parameters for different algorithms.

Algorithms	Number of Trainable Parameters			
	CCNN	CACNN	SEACNN	SACNN
**DN121**	16,180,103	16,222,695	23,067,145	22,035,367
**DN169**	18,702,983	18,745,575	25,590,025	21,412,519
**DN201**	24,336,519	24,379,111	31,223,561	27,046,055
**MN**	12,433,223	12,475,815	19,320,265	18,288,487
**MNv2**	11,456,391	11,498,983	18,343,433	17,311,655
**MNv3L**	9,121,463	9,164,055	16,008,505	11,830,999
**Iv3**	31,019,687	31,062,279	37,906,729	36,874,951
**IRv2**	63,514,983	63,557,575	70,402,025	69,370,247
**X**	30,058,287	30,100,879	36,945,329	35,913,551

Here, it’s evident that IRv2 has the highest number of parameters, with SEACNN training approximately 70 million parameters. The other models have less than half of that. Given that all algorithms operate independently and in parallel before being ensembled, the ultimate prediction by IGPA is achieved efficiently without significant time overhead.

#### 5.2.2 Hyperparameters selection

The hyperparameters were selected through a meticulous process of manual tuning guided by both empirical observations and established best practices in deep learning. Each choice, from the learning rate and batch size to the specific architecture decisions like kernel sizes and activation functions, was carefully evaluated to optimize model performance while ensuring robustness against overfitting. This approach leveraged insights gained from extensive experimentation and a deep understanding of the network’s dynamics, aiming to strike a balance between computational efficiency and achieving state-of-the-art results in the task at hand.

We utilized a learning rate of 0.001 with the Adam optimizer to support precise weight adjustments, crucial for navigating the intricate optimization landscape of our CNN. Additionally, batch normalization was implemented to stabilize training dynamics by normalizing layer inputs, thereby enhancing convergence speed and minimizing overfitting risks. The kernel initializer ‘he_normal’ ensured effective weight initialization, which in turn facilitated gradient flow maintenance and accelerated model learning capacity. Furthermore, employing the ReLU activation function enabled our model to efficiently capture complex data patterns and relationships, crucial for achieving high accuracy in classification tasks.

### 5.3 CTL architectures in Level 1

A total of nine models mentioned earlier were employed, which were paired with CCNN, CACNN, SEACNN, and SACNN for each model variant. The outcomes obtained from these diverse combinations, along with the results from level-1 IGPA, are presented in Tables 3 through 11. Both scenarios of IGPA performance using all classifiers and the best 3 classifiers are included. Specifically, IGPA with all classifiers in level ‘i’ is referred to as *IGPA*_*ai*_, while IGPA with the best 3 classifiers is denoted as *IGPA*_*bi*_.

**Table 3:** Performance metrics of DenseNet121 approaches.

Algorithm	Accuracy	Precision	Recall	F1-score	Specificity
CCNN	92.39	91.99	92.39	92.12	86.65
CACNN	91.67	91.04	91.67	91.17	82.30
SEACNN	92.03	91.46	92.03	91.43	82.80
SACNN	92.03	91.76	92.03	91.76	85.64
*IGPA*_*a*1_	93.36	93.04	93.36	92.96	84.77
*IGPA*_*b*1_	93.84	93.62	93.84	93.45	86.71

**Table 4:** Performance metrics of DenseNet169 approaches.

Algorithm	Accuracy	Precision	Recall	F1-score	Specificity
CCNN	92.75	92.54	92.75	92.39	85.75
CACNN	92.03	91.55	92.03	91.71	85.19
SEACNN	90.58	90.38	90.58	90.09	85.54
SACNN	91.67	91.54	91.67	91.22	86.57
*IGPA*_*a*1_	93.36	93.02	93.36	93.00	86.70
*IGPA*_*b*1_	93.48	93.10	93.48	93.10	86.23

**Table 5:** Performance metrics of DenseNet201 approaches.

Algorithm	Accuracy	Precision	Recall	F1-score	Specificity
CCNN	91.18	90.92	91.18	90.94	86.15
CACNN	91.79	91.24	91.79	91.20	84.24
SEACNN	92.28	92.00	92.28	91.81	82.78
SACNN	92.03	91.81	92.03	91.75	85.64
*IGPA*_*a*1_	93.36	93.10	93.36	93.05	86.22
*IGPA*_*b*1_	93.84	93.74	93.84	93.49	87.19

**Table 6:** Performance metrics of MobileNet approaches.

Algorithm	Accuracy	Precision	Recall	F1-score	Specificity
CCNN	91.42	90.90	91.42	90.86	80.86
CACNN	92.75	92.42	92.75	92.26	81.43
SEACNN	91.18	90.69	91.18	90.85	83.72
SACNN	91.67	88.85	91.67	91.46	88.49
*IGPA*_*a*1_	93.48	93.21	93.48	92.97	83.36
*IGPA*_*b*1_	93.60	93.34	93.60	93.08	84.80

**Table 7:** Performance metrics of MobileNetV2 approaches.

Algorithm	Accuracy	Precision	Recall	F1-score	Specificity
CCNN	91.90	91.80	91.90	91.60	83.31
CACNN	91.30	90.81	91.30	90.72	79.86
SEACNN	90.94	90.21	90.94	90.08	78.88
SACNN	91.42	90.91	91.42	90.54	77.98
*IGPA*_*a*1_	93.60	93.39	93.60	92.93	82.42
*IGPA*_*b*1_	93.36	93.08	93.36	92.73	81.91

**Table 8:** Performance metrics of MobileNetV3Large approaches.

Algorithm	Accuracy	Precision	Recall	F1-score	Specificity
CCNN	92.99	92.60	92.99	92.34	85.25
CACNN	91.67	91.55	91.67	91.54	88.04
SEACNN	92.14	91.61	92.14	91.48	82.79
SACNN	91.78	91.45	91.78	91.43	84.71
*IGPA*_*a*1_	94.08	93.93	94.08	93.55	86.25
*IGPA*_*b*1_	94.20	94.06	94.20	93.68	86.74

**Table 9:** Performance metrics of InceptionV3 approaches.

Algorithm	Accuracy	Precision	Recall	F1-score	Specificity
CCNN	92.27	91.86	92.27	91.62	85.19
CACNN	90.94	90.16	90.94	90.34	79.87
SEACNN	90.82	90.84	90.82	90.79	88.02
SACNN	90.82	90.51	90.82	90.60	86.60
*IGPA*_*a*1_	93.24	92.74	93.24	92.75	86.21
*IGPA*_*b*1_	93.72	93.22	93.72	93.27	87.19

**Table 10:** Performance metrics of InceptionResnetV2 approaches.

Algorithm	Accuracy	Precision	Recall	F1-score	Specificity
CCNN	90.70	89.85	90.70	90.01	79.39
CACNN	91.18	91.54	91.18	91.16	87.07
SEACNN	89.85	89.99	89.85	89.57	83.17
SACNN	91.67	91.30	91.67	91.38	86.59
*IGPA*_*a*1_	93.48	93.12	93.48	93.06	87.65
*IGPA*_*b*1_	93.60	93.12	93.60	93.20	88.15

**Table 11:** Performance metrics of Xception approaches.

Algorithm	Accuracy	Precision	Recall	F1-score	Specificity
CCNN	90.45	89.96	90.45	90.15	84.18
CACNN	90.33	89.60	90.33	89.63	82.71
SEACNN	92.51	92.04	92.51	92.08	84.26
SACNN	91.78	91.87	91.78	91.65	88.03
*IGPA*_*a*1_	93.48	93.08	93.48	93.09	86.71
*IGPA*_*b*1_	93.36	92.98	93.36	92.95	87.17

The justification for opting for the best 3 classifiers instead of using all of them is found in the enhanced performance showcased in the *IGPA*_*b*1_ scores. A comparison between the performance metrics of the finest 3 classifiers and those encompassing all the classifiers reveals a consistent trend: the top 3 classifiers consistently outperform in terms of accuracy, precision, recall, F1-score, and specificity. This compellingly signifies that concentrating on these top performers results in superior model performance. Narrowing down to the best 3 classifiers makes the model selection process more efficient and impactful. It prioritizes the most pertinent and accurate classifiers for the given task. This emphasis on the elite 3 classifiers not only elevates overall performance but also diminishes the computational intricacy and resource demands linked with utilizing the entire array of available classifiers.

### 5.4 CTL architectures in Level 2

At Level 2, our approach involves leveraging three distinctive combinations derived from the Level-1 predictions. To elucidate, the initial blend encompasses the three DenseNet models: DN121, DN169, and DN201. This amalgamated model is aptly referred to as ‘DN’ shown in Table 12.

**Table 12:** Performance metrics of DN approaches.

**Algorithm** *IGPA*_*a*1_	**Accuracy**	Precision	Recall	F1-score	Specificity
DN121	93.36	93.04	93.36	92.96	84.77
DN169	93.36	93.02	93.36	93.00	86.70
DN201	93.36	93.10	93.36	93.05	86.22
*IGPA*_*a*2_	93.72	93.41	93.72	93.39	86.72
**Algorithm** *IGPA*_*b*1_	**Accuracy**	Precision	Recall	F1-score	Specificity
DN121	93.84	93.62	93.84	93.45	86.71
DN169	93.48	93.10	93.48	93.10	86.23
DN201	93.84	93.74	93.84	93.49	87.19
*IGPA*_*b*2_	94.08	93.83	94.08	93.74	88.16

Moving on, the ‘MN’ configuration harmonizes the predictive power of MobileNet, MobileNetV2, and Xception. The performances of MN are depicted in Table 13.

**Table 13:** Performance metrics of MN approaches.

**Algorithm** *IGPA*_*a*1_	**Accuracy**	Precision	Recall	F1-score	Specificity
MN	93.48	93.21	93.48	92.97	83.36
MNv2	93.60	93.39	93.60	92.93	82.42
MNv3L	94.08	93.93	94.08	93.55	86.25
*IGPA*_*a*2_	94.32	94.19	94.32	93.78	84.36
**Algorithm** *IGPA*_*b*1_	**Accuracy**	Precision	Recall	F1-score	Specificity
MN	93.60	93.34	93.60	93.08	84.80
MNv2	93.36	93.08	93.36	92.73	81.91
MNv3L	94.20	94.06	94.20	93.68	86.74
*IGPA*_*b*2_	94.81	94.84	94.81	94.31	86.30

Lastly, the composite ‘IX’ amalgamation encapsulates the predictive prowess of InceptionV3, InceptionResnetV2, and Xception models whose outcome is decorated in Table 14.

**Table 14:** Performance metrics of IX approaches.

**Algorithm** *IGPA*_*a*1_	**Accuracy**	Precision	Recall	F1-score	Specificity
Iv3	93.24	92.74	93.24	92.75	86.21
IRv2	93.48	93.12	93.48	93.06	87.65
X	93.48	93.08	93.48	93.09	86.71
*IGPA*_*a*2_	94.20	94.10	94.20	93.78	87.69
**Algorithm** *IGPA*_*b*1_	**Accuracy**	Precision	Recall	F1-score	Specificity
Iv3	93.72	93.22	93.72	93.27	87.19
IRv2	93.60	93.12	93.60	93.20	88.15
X	93.36	92.98	93.36	92.95	87.17
*IGPA*_*b*2_	94.45	94.22	94.45	94.06	88.67

Notably, throughout this level, it is apparent that the *IGPA*_*b*2_ consistently exhibits superior performance compared to *IGPA*_*a*2_ across most instances.

### 5.5 CTL architectures in Level 3

In the ultimate stage, the strength of three predictions garnered from Level 2 is harnessed. The refined selection of these three is meticulously scrutinized, and the outcomes acquired from this final level are depicted in Table 15. Notably, in this context, it becomes overtly apparent that *IGPA*_*b*3_ consistently outperforms *IGPA*_*a*3_ across all performance evaluation metrics.

**Table 15:** Performance metrics of DMIX approaches.

**Algorithm** *IGPA*_*a*2_	**Accuracy**	Precision	Recall	F1-score	Specificity
DN	93.72	93.41	93.72	93.39	86.72
MN	94.32	94.19	94.32	93.78	84.36
IX	94.20	94.10	94.20	93.78	87.69
*IGPA*_*a*3_	94.69	94.55	94.69	94.29	86.76
**Algorithm** *IGPA*_*b*2_	**Accuracy**	Precision	Recall	F1-score	Specificity
DN	94.08	93.83	94.08	93.74	88.16
MN	94.81	94.84	94.81	94.31	86.30
IX	94.45	94.22	94.45	94.06	88.67
*IGPA*_*b*3_	**94.93**	**94.88**	**94.93**	**94.54**	**87.26**

More specifically, the ensemble variant *IGPA*_*b*3_—an amalgamation of DN, MNX, and IX from the preceding level—shines with a remarkable accuracy of 94.93%. Conversely, *IGPA*_*a*3_ demonstrates a commendable accuracy of 94.69%, employing predictions from all the previous levels. In terms of precision, recall, F1-score, and specificity, it’s worth noting that *IGPA*_*a*3_ showcases marginally lower performance compared to *IGPA*_*b*3_.

### 5.6 CTL architectures in Single-Level IGPA

In the single-level IGPA approach, the evaluation metrics are established to reassess the validity of the assertion regarding the superiority of the Multi-Level IGPA. The results, showcased in Table 16, vividly demonstrate that the multi-level approach significantly outperforms the single-level IGPA, yielding a mere 93.96% accuracy. This disparity is also substantiated by the values of other performance metrics, effectively confirming the soundness of the claim.

**Table 16:** Performance metrics of Single-Levelled IGPA.

Algorithm	Accuracy	Precision	Recall	F1-score	Specificity
*IGPA*	93.96	93.70	93.96	93.87	85.35

### 5.7 Performance analysis by visualization

#### 5.7.1 Confusion matrix

**Fig 9:**
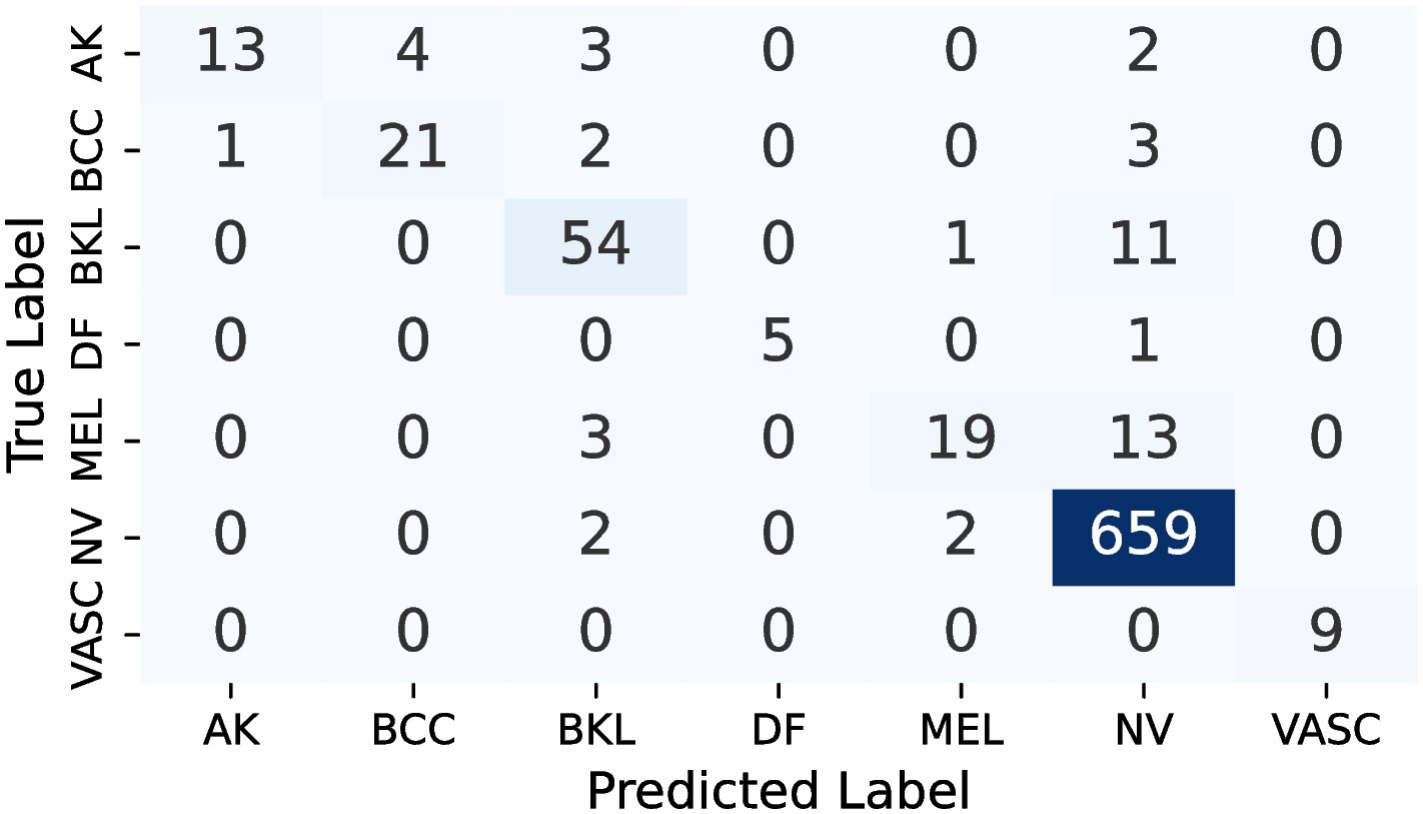
Confusion matrix obtained by SL-IGPA.

**Fig 10:**
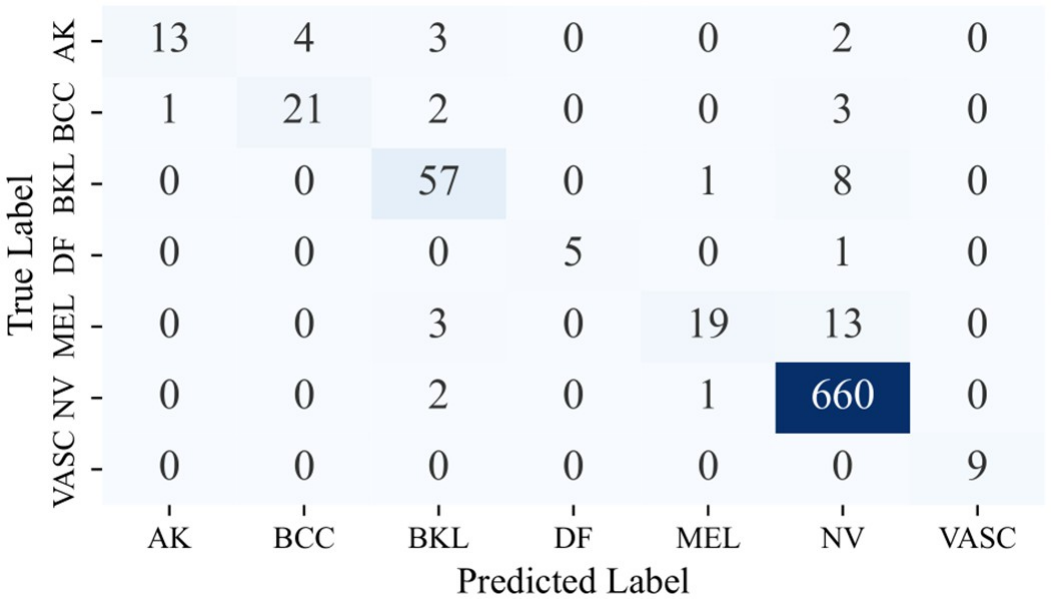
Confusion matrix obtained by ML-IGPA (All classifiers).

**Fig 11:**
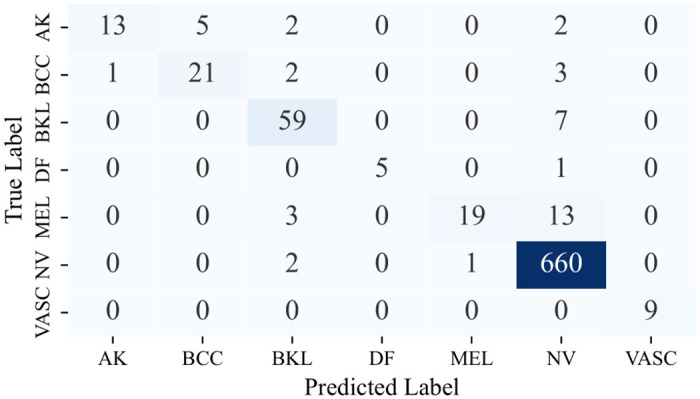
Confusion matrix obtained by ML-IGPA (Best 3 classifiers).

Owing to the utilization of a comprehensive range of classifiers, each with its distinct variations, the decision was made to refrain from presenting the confusion matrices for each. Instead, the focus is on showcasing the confusion matrices stemming from the Multi-Level IGPA approach employing both the complete set of classifiers and the top 3 classifiers, along with the Single-Level IGPA. These visual representations, as seen in Figs 9–11, offer a clear depiction of the correct and incorrect classification rates for each category. Moreover, they validate that the ML-IGPA surpasses the SL-IGPA in sample classification. Notably, it becomes apparent that the ML-IGPA, when utilized with the top 3 classifiers, results in fewer instances of misclassification compared to its use with all classifiers.

More specifically, the performance of our proposed *ML*−*IGPA*_*b*_3 architecture across different classes highlights its effectiveness. The VASC class saw perfect accuracy, with all 9 samples correctly classified. For the DF class, 5 out of 6 samples were accurately classified, with only one misclassification. The NV class showcased remarkable performance, correctly classifying 660 out of 663 samples, indicating strong performance in both minority and majority classes. For the AK class, 13 samples were correctly identified with 9 misclassifications, while the BCC class saw 21 correct classifications and 6 errors. The BKL class also performed well, with 59 out of 66 samples correctly classified. The MEL class, although the most challenging, still achieved 19 correct classifications out of 35 samples. Overall, our architecture not only performs exceptionally well but demonstrates significant accuracy across all classes, solidifying its robustness and reliability.

#### 5.7.2 Receiver Operating Characteristic Area Under Curve (ROC-AUC)

**Fig 12:**
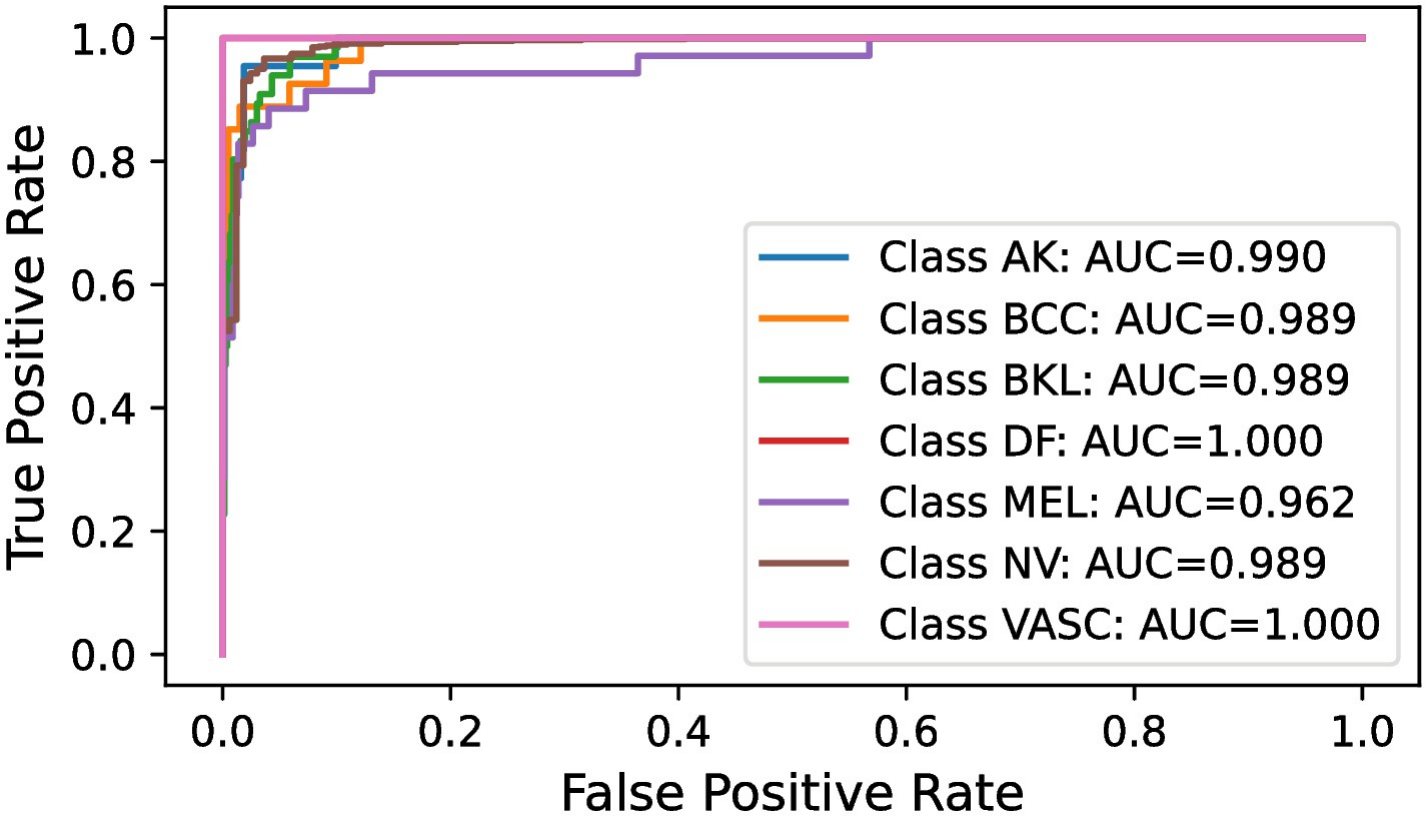
ROC-AUC curve obtained by SL-IGPA.

**Fig 13:**
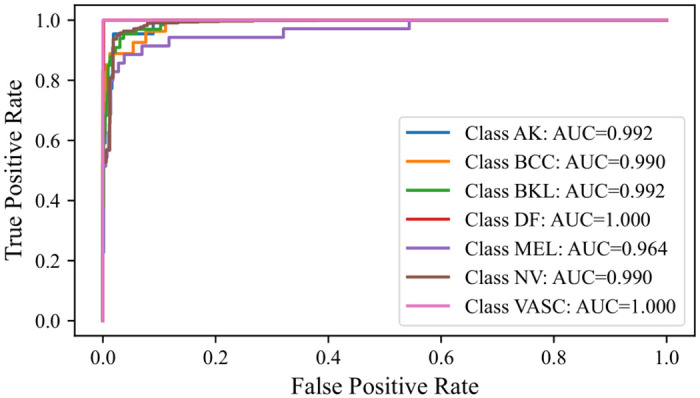
ROC-AUC curve obtained by ML-IGPA (All classifiers).

**Fig 14:**
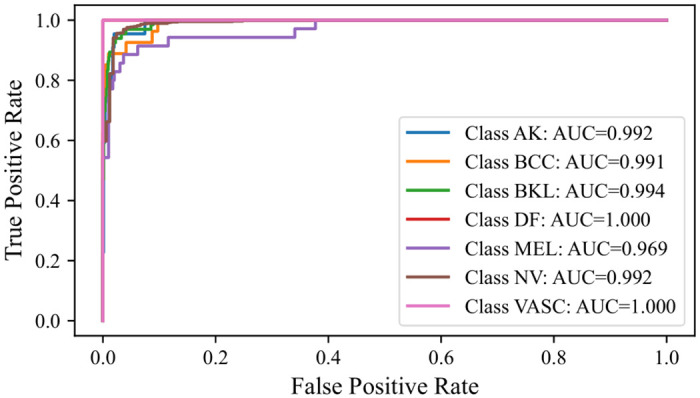
ROC-AUC curve obtained by ML-IGPA (Best 3 classifiers).

The ROC-AUC curve effectively visualizes a model’s performance. Consequently, an analysis of this metric was conducted. The ROC-AUC curves for the Multi-Level IGPA approach, using both the complete set of classifiers and the top 3 classifiers, alongside the Single-Level IGPA, were graphically represented. As with the rationale behind excluding the extensive number of classifiers for the confusion matrix, a similar approach was adopted for the ROC-AUC curves. Hence, they are showcased in Figs 12–14. These curves highlight a significant observation: the ROC-AUC curve derived from the Multi-Level IGPA utilizing the best 3 classifiers displays reduced fluctuations compared to the others. Moreover, the curve for Multi-Level IGPA with all classifiers demonstrates superior performance compared to the Single-Level IGPA. Overall, these results substantiate the validity of our assertion.

#### 5.7.3 Gradient Class Activation Map (GradCAM)

**Fig 15:**
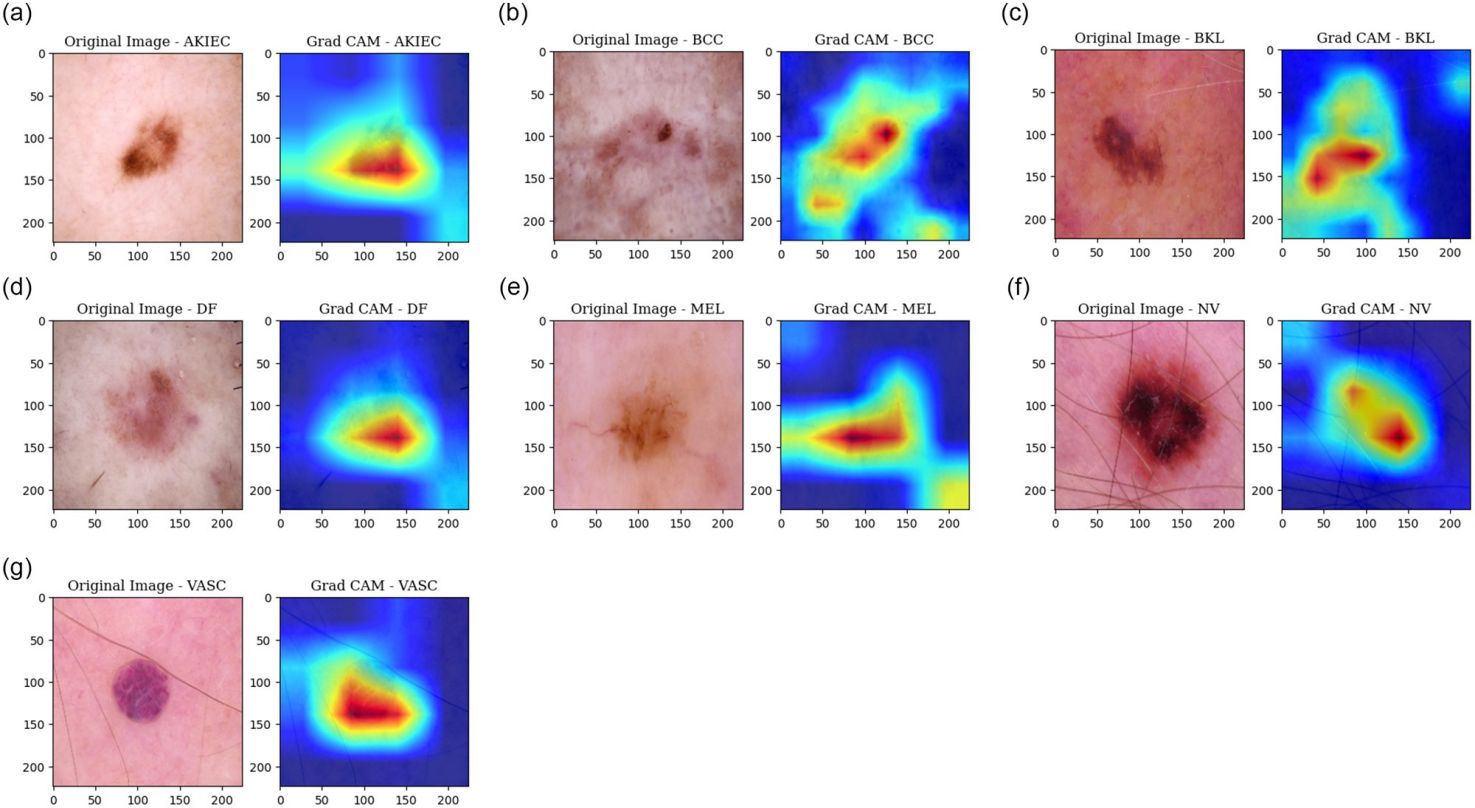
GradCAM generation by the model for each class. (a) GradCAM for AK, (b) GradCAM for BCC, (c) GradCAM for BKL, (d) GradCAM for DF, (e) GradCAM for MEL, (f) GradCAM for NV, (g) GradCAM for VASC.

To create the visualization, the process begins by generating a heatmap from the original image. This heatmap highlights the areas of focus determined by the model. Subsequently, the GradCAM view is derived from this heatmap, offering a detailed depiction of the regions that the model prioritizes within the image. The visualization for each class is illustrated in Fig 15, where seven instances from seven classes are taken and then the corresponding GradCAM view with the original image is integrated. Our model excels at identifying the precise regions within each image that are of greater significance, rather than processing the entire image, which significantly enhances its ability to accurately classify them.

**Fig 16:**
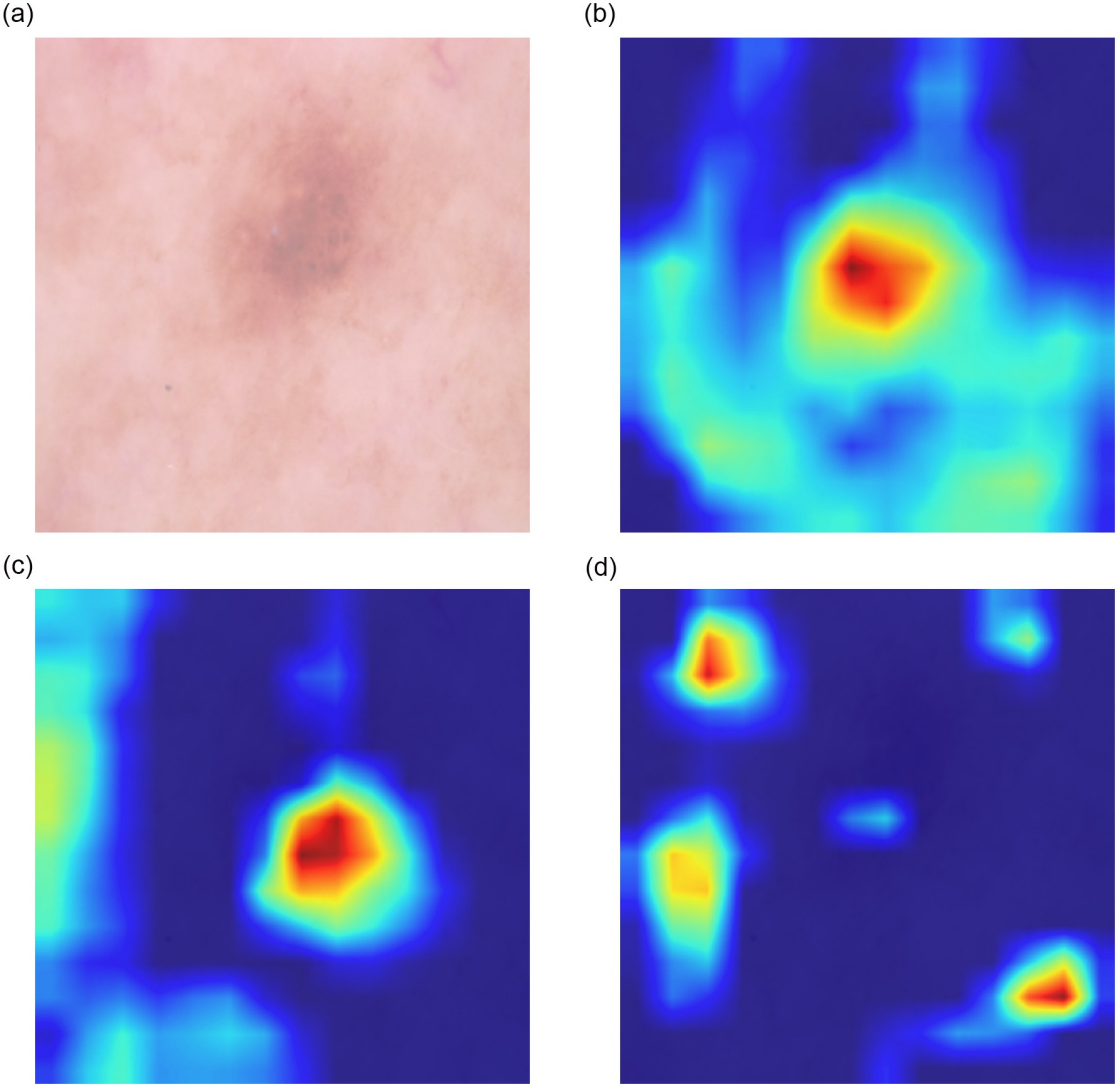
GradCAM visualization for architecture explainability (Example by DN121). (a) Original, (b) CACNN, (c) SEACNN, (d) SACNN.

When a model is capable of generating an accurate heatmap that covers the relevant region, it indicates that the model can make correct classifications. Conversely, if the heatmap is incorrect, it implies that the model’s classification might also be inaccurate. To illustrate this concept, let’s consider an example for better comprehension in Fig 16.

In subfigure Fig 16, the original image stands as an exemplar. Remarkably, by DenseNet121, the CA and SEA-based CNNs accurately predict the image’s correct class, yet the SA-based CNN falls short in this aspect. Similarly, the involvement of other models corroborates our assertion. Through the amalgamation of multiple models, our final prediction consistently emerges as accurate. This fact becomes evident when examining the GradCAM visualizations of these other models, which further solidifies our contention. However, an ingenious approach comes into play with the IGPA ensemble, allowing us to successfully predict the accurate class. This observation further underscores the prowess of our advanced Multi-Level IGPA technique, showcasing its capacity to overcome individual classifier limitations and validate its superiority in achieving accurate predictions.

### 5.8 Ablation study

To demonstrate the superiority of our novel approach compared to state-of-the-art methods, we conducted a comprehensive ablation study focusing on two key innovations: Triple Attention (TA) and Information Gain Proportioned Averaging (IGPA). We evaluated the performance impact of these components by analyzing the results with and without their utilization.

#### 5.8.1 Utilization of IGPA without TA

We applied IGPA across various models, including DenseNet, MobileNet, and Inception, at different levels as previously mentioned. Each model was tested in four configurations: three with TA and one without attention modules. To highlight the efficacy of TA, we present the results in Table 17, showcasing the performance of IGPA in both Multi-Level Ensemble (MLE) and Single Level Ensemble (SLE) setups, excluding the TA-integrated models.

**Table 17:** Performance metrics of IGPA without TA.

Algorithm	Accuracy	Precision	Recall	F1-score	Specificity
DN121_CCNN	92.39	91.99	92.39	92.12	86.65
DN169_CCNN	92.75	92.54	92.75	92.39	85.75
DN201_CCNN	91.18	90.92	91.18	90.94	86.15
*IGPA*_*DN*_	93.84	93.55	93.84	93.56	86.28
MN_CCNN	91.42	90.90	91.42	90.86	80.86
MNv2_CCNN	91.90	91.80	91.90	91.60	83.31
MNv3L_CCNN	92.99	92.60	92.99	92.34	85.25
*IGPA*_*MN*_	93.24	93.16	93.24	92.66	83.35
Iv3_CCNN	92.27	91.86	92.27	91.62	85.19
IRv2_CCNN	90.70	89.85	90.70	90.01	79.39
X_CCNN	90.45	89.96	90.45	90.15	84.18
*IGPA*_*IX*_	92.15	91.48	92.15	91.56	83.77
*IGPA*_*MLE*_	93.48	93.20	93.48	92.91	83.26
*IGPA*_*SLE*_	93.48	93.98	93.48	93.80	85.30
**Ours**	**94.93**	**94.88**	**94.93**	**94.54**	**87.26**

We compared the performance of our complete proposed architecture with and without the integration of TA (Triple Attention). Our results clearly demonstrate that omitting TA significantly degrades performance. Specifically, at each level, models without TA underperform compared to those with integrated TA. Notably, without TA, both *IGPA*_*MLE*_ and *IGPA*_*SLE*_ achieve 93.48% accuracy. In contrast, our TA-integrated IGPA achieves a higher accuracy of 94.93%, along with improvements in other metrics.

#### 5.8.2 Utilization of conventional ensemble methods instead of IGPA

As previously mentioned, we have applied IGPA at multiple levels using two distinct approaches. Predictions from CCNN, CACNN, SEACNN, and SACNN models are ensembled by determining the optimal weights for all models as well as for the top three models. Specifically, IGPA utilizing all classifiers at level *i* is denoted as *IGPA*_*ai*_, while IGPA employing the top three classifiers is labeled as *IGPA*_*bi*_. To demonstrate the superiority of IGPA, we compared its performance against traditional ensemble methods, including Softmax Averaging (SA), Majority Voting (MV), and Weighted Averaging (WA) with random weights. The results of these comparisons are presented here.

**Softmax Averaging (SA)**: As shown in following tables, Softmax Averaging (SA) using all classifiers at level *i* is denoted as *SA*_*ai*_, while SA employing the top three classifiers is labeled as *SA*_*bi*_ and the last table presents a performance comparison of single-level ensembles.

In Table 18, for instance, the *DMIX*_*IGPA*_*a*3_ model achieves an accuracy of 94.69%, significantly higher than the best-performing non-IGPA model, which reaches 94.20%. This trend continues in Table 19, where the *DMIX*_*IGPA*_*b*3_ model achieves the highest accuracy of 94.93%, compared to the best non-IGPA model’s 94.32%.

**Table 18:** Performance metrics of Softmax Averaging of all classifiers.

Algorithm	Accuracy	Precision	Recall	F1-score	Specificity
*DN*121_*SA*_*a*1_	92.63	92.18	92.63	92.17	82.34
*DN*169_*SA*_*a*1_	92.63	92.18	92.63	92.21	85.69
*DN*201_*SA*_*a*1_	93.24	92.89	93.24	92.90	85.75
*DN*_*SA*_*a*2_	93.36	92.97	93.36	93.00	85.74
*MN*_*SA*_*a*1_	93.00	92.58	93.00	92.46	82.38
*MNv*2_*SA*_*a*1_	93.48	93.26	93.48	92.80	81.94
*MNv*3*L*_*SA*_*a*1_	93.48	93.19	93.48	92.92	84.79
*MN*_*SA*_*a*2_	93.96	93.69	93.96	93.40	83.38
*Iv*3_*SA*_*a*1_	92.75	92.15	92.75	92.26	85.22
*IRv*2_*SA*_*a*1_	93.00	92.56	93.00	92.55	86.19
*X*_*SA*_*a*1_	92.63	92.14	92.63	92.15	84.26
*IX*_*SA*_*a*2_	93.60	93.30	93.60	93.15	86.23
*DMIX*_*SA*_*a*3_	94.20	93.98	94.20	93.80	85.30
*DMIX*_*IGPA*_*a*3_	94.69	94.55	94.69	94.29	86.76

**Table 19:** Performance metrics of Softmax Averaging of best 3 classifiers.

Algorithm	Accuracy	Precision	Recall	F1-score	Specificity
*DN*121_*SA*_*b*1_	93.00	92.67	93.00	92.56	83.79
*DN*169_*SA*_*b*1_	92.87	92.43	92.87	92.44	83.80
*DN*201_*SA*_*b*1_	93.24	92.99	93.24	92.85	85.25
*DN*_*SA*_*b*2_	93.48	93.12	93.48	93.11	86.12
*MN*_*SA*_*b*1_	92.75	92.36	92.75	92.24	85.33
*MNv*2_*SA*_*b*1_	93.00	92.69	93.00	92.34	80.94
*MNv*3*L*_*SA*_*b*1_	93.48	93.17	93.48	92.91	84.30
*MN*_*SA*_*b*2_	94.20	94.07	94.20	93.68	84.35
*Iv*3_*SA*_*b*1_	93.12	92.60	93.12	92.71	85.73
*IRv*2_*SA*_*b*1_	92.87	92.31	92.87	92.45	86.20
*X*_*SA*_*b*1_	92.51	91.99	92.51	92.05	84.26
*IX*_*SA*_*b*2_	93.60	93.21	93.60	93.19	86.71
*DMIX*_*SA*_*b*3_	94.32	94.16	94.32	93.91	85.31
*DMIX*_*IGPA*_*b*3_	**94.93**	**94.88**	**94.93**	**94.54**	**87.26**

Furthermore, Table 20 highlights the performance of single-level ensembles, where the SL_IGPA model outperforms the SL_SA model with an accuracy of 93.96% compared to 93.48%. These results clearly demonstrate that the integration of IGPA leads to superior performance across multiple evaluation metrics, establishing our IGPA-integrated models as the more effective approach.

**Table 20:** Performance metrics of Single Level Softmax Averaging.

Algorithm	Accuracy	Precision	Recall	F1-score	Specificity
SL_SA	93.48	93.18	93.48	92.91	83.35
SL_IGPA	93.96	93.70	93.96	93.87	85.35

Thus, it is evident that the highest accuracy obtained by *SA*_*b*3_ is 0.61% lower than that of our *IGPA*_*b*3_.

**Majority Voting (MV)**: As shown in the upcoming tables, Majority Voting (MV) using all classifiers at level *i* is denoted as *MV*_*ai*_, while MV employing the top three classifiers is labeled as *MV*_*bi*_ and also the last table presents a performance comparison of single-level ensembles.

In Table 21, the *DMIX*_*IGPA*_*a*3_ model achieves an accuracy of 94.69%, significantly higher than the best-performing non-IGPA model, which reaches 93.96%. This trend continues in Table 22, where the *DMIX*_*IGPA*_*b*3_ model achieves the highest accuracy of 94.93%, compared to the best non-IGPA model’s 94.08%.

**Table 21:** Performance metrics of Majority Voting of all classifiers.

Algorithm	Accuracy	Precision	Recall	F1-score	Specificity
*DN*121_*MV*_*a*1_	93.24	92.91	93.24	92.89	86.67
*DN*169_*MV*_*a*1_	93.72	93.47	93.72	93.49	89.59
*DN*201_*MV*_*a*1_	92.87	92.51	92.87	92.61	87.15
*DN*_*MV*_*a*2_	93.36	93.09	93.36	93.08	88.12
*MN*_*MV*_*a*1_	92.75	92.72	92.75	92.34	84.28
*MNv*2_*MV*_*a*1_	93.60	93.28	93.60	93.13	84.80
*MNv*3*L*_*MV*_*a*1_	93.48	93.48	93.48	93.13	88.61
*MN*_*MV*_*a*2_	94.44	94.22	94.44	94.02	86.76
*Iv*3_*MV*_*a*1_	92.75	92.79	92.75	92.42	87.63
*IRv*2_*MV*_*a*1_	93.00	92.98	93.00	92.71	88.57
*X*_*MV*_*a*1_	92.63	92.21	92.63	92.26	85.71
*IX*_*MV*_*a*2_	93.72	93.72	93.72	93.40	88.63
*DMIX*_*MV*_*a*3_	93.96	93.96	93.96	93.55	87.20
*DMIX*_*IGPA*_*a*3_	94.69	94.55	94.69	94.29	86.76

**Table 22:** Performance metrics of Majority Voting of best 3 classifiers.

Algorithm	Accuracy	Precision	Recall	F1-score	Specificity
*DN*121_*MV*_*b*1_	92.87	92.58	92.87	92.45	85.71
*DN*169_*MV*_*b*1_	93.12	93.12	93.12	92.76	85.75
*DN*201_*MV*_*b*1_	92.75	92.75	92.75	92.32	84.27
*DN*_*MV*_*b*2_	93.24	93.24	93.24	92.87	86.21
*MN*_*MV*_*b*1_	92.87	92.46	92.87	92.36	83.83
*MNv*2_*MV*_*b*1_	92.75	92.75	92.75	92.24	82.37
*MNv*3*L*_*MV*_*b*1_	93.48	93.48	93.48	92.97	85.26
*MN*_*MV*_*b*2_	93.84	93.84	93.84	93.28	84.34
*Iv*3_*MV*_*b*1_	92.87	92.25	92.87	92.46	87.16
*IRv*2_*MV*_*b*1_	92.87	92.38	92.87	92.53	86.21
*X*_*MV*_*b*1_	92.63	92.63	92.63	92.29	85.70
*IX*_*MV*_*b*2_	94.08	93.96	94.08	93.74	88.66
*DMIX*_*MV*_*b*3_	94.08	93.86	94.08	93.71	86.77
*DMIX*_*IGPA*_*b*3_	**94.93**	**94.88**	**94.93**	**94.54**	**87.26**

Furthermore, Table 23 highlights the performance of single-level ensembles, where the SL_IGPA model outperforms the SL_MV model with an accuracy of 93.96% compared to 93.72%. These results clearly demonstrate that the integration of IGPA leads to superior performance across multiple evaluation metrics, establishing our IGPA-integrated models as the more effective approach.

**Table 23:** Performance metrics of Single Level Majority Voting.

Algorithm	Accuracy	Precision	Recall	F1-score	Specificity
SL_MV	93.72	93.72	93.72	93.24	84.32
SL_IGPA	93.96	93.70	93.96	93.87	85.35

Thus, it is clear that the highest accuracy achieved by *MV*_*b*3_ is 0.85% lower than that of our *IGPA*_*b*3_.

**Weighted Averaging (WA)**: As depicted in the next two tables, Weighted Averaging (WA) with all classifiers at level *i* is denoted as *WA*_*ai*_, while WA with the best three classifiers is labeled as *WA*_*bi*_. We utilized random weights for each classifier: in the ensemble of all four classifiers, we assigned 30% weight to the best-performing algorithm, followed by 26%, 24%, and 20% for the least performing. For the ensemble of the top three classifiers, we assigned 35% weight to the top two classifiers and 30% to the third. The final table presents a performance comparison of single-level ensembles.

Table 24 shows the performance metrics of Weighted Averaging of all classifiers (*WA*_*ai*_). The highest accuracy achieved is 94.20% by the *DMIX*_*WA*_*a*3_ algorithm. However, the highest-performing algorithm using IGPA, *DMIX*_*IGPA*_*a*3_, achieves an accuracy of 94.69%, which is significantly higher. Additionally, the IGPA method consistently outperforms WA in all other metrics: precision, recall, F1-score, and specificity. For instance, *DMIX*_*IGPA*_*a*3_ has a precision of 94.55% compared to 94.20% for *DMIX*_*WA*_*a*3_, highlighting the superior performance of IGPA.

**Table 24:** Performance metrics of Weighted Averaging of all classifiers.

Algorithm	Accuracy	Precision	Recall	F1-score	Specificity
*DN*121_*WA*_*a*1_	92.27	91.93	92.27	91.78	82.32
*DN*169_*WA*_*a*1_	92.87	92.45	92.87	92.47	84.77
*DN*201_*WA*_*a*1_	92.87	92.49	92.87	92.48	84.28
*DN*_*WA*_*a*2_	93.48	93.11	93.48	93.15	85.75
*MN*_*WA*_*a*1_	92.87	92.48	92.87	92.34	82.38
*MNv*2_*WA*_*a*1_	93.36	93.36	93.36	92.66	80.97
*MNv*3*L*_*WA*_*a*1_	93.48	93.17	93.48	92.91	84.30
*MN*_*WA*_*a*2_	94.08	93.99	94.08	93.51	83.86
*Iv*3_*WA*_*a*1_	93.00	92.99	93.00	92.50	85.70
*IRv*2_*WA*_*a*1_	92.87	92.85	92.87	92.43	86.18
*X*_*WA*_*a*1_	92.63	92.63	92.63	92.19	84.26
*IX*_*WA*_*a*2_	93.84	93.84	93.84	93.43	86.72
*DMIX*_*WA*_*a*3_	94.20	94.20	94.20	93.80	85.30
*DMIX*_*IGPA*_*a*3_	94.69	94.55	94.69	94.29	86.76

Table 25 presents the performance metrics of Weighted Averaging of the best three classifiers (*WA*_*bi*_). The best accuracy using WA is 94.32% by the *DMIX*_*WA*_*b*3_ algorithm. In contrast, the *DMIX*_*IGPA*_*b*3_ algorithm using IGPA achieves an even higher accuracy of 94.93%. The improvement in performance is also seen across all other metrics. For example, *DMIX*_*IGPA*_*b*3_ has a precision of 94.88%, recall of 94.93%, F1-score of 94.54%, and specificity of 87.26%, all of which surpass the corresponding values for *DMIX*_*WA*_*b*3_.

**Table 25:** Performance metrics of Weighted Averaging of best 3 classifiers.

Algorithm	Accuracy	Precision	Recall	F1-score	Specificity
*DN*121_*WA*_*b*1_	92.87	92.41	92.87	92.48	84.75
*DN*169_*WA*_*b*1_	92.75	92.75	92.75	92.32	83.80
*DN*201_*WA*_*b*1_	93.24	93.24	93.24	92.85	85.25
*DN*_*WA*_*b*2_	93.48	93.48	93.48	93.11	86.22
*MN*_*WA*_*b*1_	92.63	92.63	92.63	92.06	82.36
*MNv*2_*WA*_*b*1_	93.00	92.99	92.99	92.34	80.94
*MNv*3*L*_*WA*_*b*1_	93.48	93.47	93.48	92.88	84.78
*MN*_*WA*_*b*2_	94.08	93.94	94.08	93.55	84.34
*Iv*3_*WA*_*b*1_	92.75	92.14	92.75	92.26	84.75
*IRv*2_*WA*_*b*1_	92.87	92.29	92.87	92.47	86.68
*X*_*WA*_*b*1_	92.39	91.86	92.39	91.94	84.25
*IX*_*WA*_*b*2_	93.84	93.49	93.84	93.45	87.21
*DMIX*_*WA*_*b*3_	94.32	94.16	94.32	93.91	85.31
*DMIX*_*IGPA*_*b*3_	**94.93**	**94.88**	**94.93**	**94.54**	**87.26**

Table 26 compares the performance of Single Level Weighted Averaging (SL_WA) with IGPA (SL_IGPA). The SL_IGPA method achieves an accuracy of 93.96%, significantly higher than the 92.15% accuracy of SL_WA. Similarly, SL_IGPA shows better performance in precision (93.70% vs. 91.50%), recall (93.96% vs. 92.15%), F1-score (93.87% vs. 91.46%), and specificity (85.35% vs. 81.87%).

**Table 26:** Performance metrics of Single Level Weighted Averaging.

Algorithm	Accuracy	Precision	Recall	F1-score	Specificity
SL_WA	92.15	91.50	92.15	91.46	81.87
SL_IGPA	93.96	93.70	93.96	93.87	85.35

Across all tables and metrics, IGPA demonstrates superior performance compared to Weighted Averaging with random weights. The consistent improvement in accuracy, precision, recall, F1-score, and specificity across different classifier ensembles proves that IGPA is a more effective method for combining classifier outputs, resulting in better overall model performance. This conclusively shows that the IGPA approach is far better than the conventional weighted averaging technique with random weights. So, It’s clear from our findings that *WA*_*b*3_ achieves an accuracy that is 0.61% lower than our *IGPA*_*b*3_.

Based on the comprehensive performance comparisons mentioned above, it is evident that our approach, integrating Triple Attention and Information Gain Proportioned Averaging, represents an optimal architecture compared to existing methods.

### 5.9 Answers to the research questions

***Answer to RQ1***: Achieving a balanced distribution of classes and generating an optimal dataset for skin lesion classification involves the implementation of data augmentation for the minority class samples. This approach aids the CTL models in learning distinguishable features during training, potentially leading to better generalization on unseen test data. Not augmenting the minority class samples during training might result in a bias towards the majority class during testing. Hence, nearly 8000 images per class are generated using data augmentation solely for the training dataset, not for validation and testing data.

***Answer to RQ2***: Emphasizing critical features, such as significant areas or regions, is realized through the utilization of Triple Attention (TA) when crafting a CNN model. It ensures that essential features receive more attention when passing feature maps from layer to layer, aiding the model in capturing relevant information. Neglecting deep features due to a simple model or overfitting training data with a complex model are risks to be balanced. Integrating TA in the CTL architecture outperforms the model without any attention mechanism, enhancing performance.

***Answer to RQ3***: The ensemble of multiple classifiers proves more effective for skin lesion classification than a single classifier, as observed in our study. The customized CNN architecture incorporates three distinct attention mechanisms (CA, SEA, and SA) separately and is associated with the TL models to construct CTL architectures. Testing conducted with multiple models recognizing unseen data, and the use of diverse ensemble strategies for predictions, demonstrates substantial improvement over using a single classifier. Utilizing an ensemble of multiple classifiers improves accuracy and the robustness of skin lesion classification models, employing diverse approaches to feature extraction and classification.

***Answer to RQ4***: The proposed Ensemble Learning approach addresses the limitations of existing techniques by dynamically calculating optimal weight ratios for each model. This data-driven method improves generalization and performance on unseen data by incorporating the best ratio of predictions from each model. The visual representation indicates substantial enhancements in performances achieved from SL-IGPA, ML-IGPA (All classifiers), and ML-IGPA (Best 3 classifiers). This indicates improved robustness and efficiency in handling diverse patterns for complex machine learning tasks.

## 6 Discussion and extended comparison

Our investigation into IGPA spans three distinct angles: Multi-Level IGPA using all classifiers, Multi-Level IGPA employing the top three classifiers, and Single-Level IGPA integrating all classifiers. We utilized various models, such as DenseNet variants, MobileNet variations, Inception, and Xception models. These were paired with different CNN architectures, resulting in nine distinct classifiers, including CCNN, CACNN, SEACNN, and SACNN. These combinations were extensively assessed at each level. The comparison between IGPA scenarios, one using all classifiers and the other selecting the top 3 classifiers (*IGPA*_*ai*_ and *IGPA*_*bi*_), highlights the significant impact of concentrating on elite classifiers. Choosing only the top 3 enhances performance across various metrics, evidenced in the *IGPA*_*b*1_ scores.

Focusing on the top 3 classifiers streamlines model selection, enhancing overall performance while reducing computational complexity. Subsequently, three combinations—DN, MN, and IX—build on the strength of each ensemble, refining the final predictions at each level. At the final level, combining Level-2 predictions highlights the consistent superiority of using the best 3 classifiers (*IGPA*_*b*3_) over all classifiers (*IGPA*_*a*3_). *IGPA*_*b*3_ reaches a remarkable 94.93% accuracy, outshining *IGPA*_*a*3_ across precision, recall, F1-score, and specificity metrics. Comparing Multi-Level and Single-Level IGPA further proves the dominance of the Multi-Level approach. Visualizations, such as confusion matrices and ROC-AUC curves, affirm the superiority of Multi-Level IGPA, especially with the best 3 classifiers, demonstrating reduced misclassification and consistent performance across various evaluation measures. Our Multi-Level IGPA with the best 3 classifiers stands out for its robustness, hierarchical approach, selective classifier use, and meticulous ensemble strategy, producing superior outcomes. Despite a larger array of classifiers, our advanced yet user-friendly approach demonstrates clear superiority in overall performance, generalization, and evaluation measures in this domain. A comprehensive comparison of our proposed model’s performance against existing literature is detailed in Table 27. Notably, we let alone compare the studies conducted by utilizing HAM10000 dataset.

**Table 27:** Comparison of our proposed model with other existing models.

Article	Accuracy	Precision	Recall	F1-Score	Specificity
[6]	84.30	-	-	-	-
[17]	86.67	-	-	-	-
[11]	94.00	-	-	-	-
[12]	91.51	-	-	-	-
[13]	80.00	-	-	-	-
[14]	91.51	-	-	-	-
[15]	86.33	-	86.33	-	97.48
[16]	89.50	-	89.50	-	**98.10**
[19]	86.20	91.30	-	-	-
[20]	88.00	87.00	94.00	89.00	-
[21]	91.24	83.53	95.04	88.91	-
[22]	93.46	-	-	-	92.90
[23]	93.46	87.01	85.57	86.28	-
[24]	86.20	-	-	-	-
[25]	83.20	-	-	-	-
[26]	94.45	93.57	94.01	94.45	-
[27]	90.00	86.00	81.00	86.00	-
[28]	93.40	93.7	-	-	-
**Ours**	**94.93**	**94.88**	**94.93**	**94.54**	87.26

## 7 Threats To validity

Listed below are certain aspects that could be considered minor limitations within our study. These aspects could potentially serve as areas for further investigation and refinement:

### 7.1 Utilization of single dataset

The study is constrained by the use of a single dataset for training and evaluation. This limitation might affect the claim of generalization of our model to diverse datasets with varying characteristics. A model trained on a single dataset might not capture the full spectrum of variations present in different sources, potentially leading to reduced performance on new and unseen data.

### 7.2 Use of a large number of classifiers in the initial level

While employing a diverse set of classifiers in the initial level can enhance the robustness of our model, it also introduces a computational burden. The use of a large number of classifiers increases the computational complexity and resource requirements during both training and inference. This could limit the scalability of our approach, especially when dealing with larger datasets or constrained computing environments.

### 7.3 Neglecting metadata of skin lesion

Our approach focuses solely on utilizing the image data of skin lesions for classification. We do not incorporate any additional metadata, such as patient demographics, lesion history, or clinical context, which could provide valuable insights for improving classification accuracy. Neglecting such supplementary information may result in missed opportunities to enhance the model’s predictive capabilities. These limitations highlight areas where our approach could be further refined and extended to address potential challenges and improve its overall performance and applicability.

## 8 Conclusion and future work

This paper addresses a critical gap in the field of ensemble learning, specifically in the context of DL methodologies. The absence of a technique that ensures optimal weight allocation for model predictions has led the research community to seek innovative solutions. A pioneering approach is introduced with the core objective of introducing a novel method termed ‘Information Gain Proportioned Averaging (IGPA).’ This method calculates the information gain associated with each model’s prediction and leverages it to determine the optimal weights for aggregating model contributions, culminating in a robust outcome.

The significance of this work extends to the domain of dermatology, where skin lesions and diseases are common health concerns. Differentiating between these conditions and pinpointing the precise regions responsible for abnormalities is pivotal. Early detection and automated predictions hold substantial value, and this study’s proposed approach addresses these challenges. By integrating a CNN-based methodology with the CTL model, a TA module, and ML-IGPA ensembling, the research achieves a remarkable advancement in skin lesion classification, surpassing existing state-of-the-art methodologies. Furthermore, the introduction of the GradCAM visualization method enhances the interpretability of the model’s outcomes. This visualization method aids in identifying the responsible regions for detecting skin lesions, thereby bridging the gap between accurate predictions and explainability.

Overall, by contributing to the early diagnosis of skin conditions and minimizing the consequences of neglect, this study bears the potential to make a positive impact on healthcare outcomes. The approach’s accessibility and cost-effectiveness further contribute to its practical applicability. As a result, this study’s findings are poised to raise awareness, transform diagnostic practices, and pave the way for improved patient care in the realm of dermatology. Looking ahead to the future, our attention will be directed towards refining the proposed model into a more streamlined version. Additionally, we intend to enhance the model’s practicality by creating a web-based API platform. This platform will enable users to submit skin images as input and receive corresponding predictions as output.
